# *Oryza glumaepatula*: A wild relative to improve drought tolerance in cultivated rice

**DOI:** 10.1093/plphys/kiad485

**Published:** 2023-09-04

**Authors:** Parthiban Thathapalli Prakash, Dmytro Chebotarov, Jianwei Zhang, David A Kudrna, Rolando O Torres, Mignon A Natividad, Marinell R Quintana, Jiaming Song, Carlos E Maldonado, Sherry Lou Hechanova, Kshirod Jena, Rod A Wing, Amelia Henry

**Affiliations:** Rice Breeding Innovations Department, International Rice Research Institute, UPLB Campus, Los Baños, Laguna 4031, Philippines; Department of Crop Sciences, University of Illinois at Urbana-Champaign, Urbana, IL 61801, USA; Rice Breeding Innovations Department, International Rice Research Institute, UPLB Campus, Los Baños, Laguna 4031, Philippines; Arizona Genomics Institute, School of Plant Sciences, University of Arizona, Tucson, AZ 85721, USA; National Key Laboratory of Crop Genetic Improvement, Hubei Hongshan Laboratory, Huazhong Agricultural University, Wuhan 430070, China; Arizona Genomics Institute, School of Plant Sciences, University of Arizona, Tucson, AZ 85721, USA; Rice Breeding Innovations Department, International Rice Research Institute, UPLB Campus, Los Baños, Laguna 4031, Philippines; Rice Breeding Innovations Department, International Rice Research Institute, UPLB Campus, Los Baños, Laguna 4031, Philippines; Rice Breeding Innovations Department, International Rice Research Institute, UPLB Campus, Los Baños, Laguna 4031, Philippines; Arizona Genomics Institute, School of Plant Sciences, University of Arizona, Tucson, AZ 85721, USA; Arizona Genomics Institute, School of Plant Sciences, University of Arizona, Tucson, AZ 85721, USA; Rice Breeding Innovations Department, International Rice Research Institute, UPLB Campus, Los Baños, Laguna 4031, Philippines; Rice Breeding Innovations Department, International Rice Research Institute, UPLB Campus, Los Baños, Laguna 4031, Philippines; School of Biotechnology, KIIT University, Bhubaneswar 751024, Odisha, India; Arizona Genomics Institute, School of Plant Sciences, University of Arizona, Tucson, AZ 85721, USA; Center for Desert Agriculture, King Abdullah University of Science & Technology, Thuwal 23955-6900, Kingdom of Saudi Arabia; Rice Breeding Innovations Department, International Rice Research Institute, UPLB Campus, Los Baños, Laguna 4031, Philippines

## Abstract

Developing drought-resistant rice (*Oryza sativa*, L.) is essential for improving field productivity, especially in rain-fed areas affected by climate change. Wild relatives of rice are potential sources for drought-resistant traits. Therefore, we compared root growth and drought response among 22 wild *Oryza* species, from which *Oryza glumaepatula* was selected as a promising source for further exploration. A geographically diverse panel of 69 *O. glumaepatula* accessions was then screened for drought stress-related traits, and 6 of these accessions showed lower shoot dry weight (SDW) reduction, greater percentage of deep roots, and lower stomatal density (STO) under drought than the drought tolerant *O. sativa* variety, Sahbhagi dhan. Based on whole-genome resequencing of all 69 *O. glumaepatula* accessions and variant calling to a high-quality *O. glumaepatula* reference genome, we detected multiple genomic loci colocating for SDW, root dry weight at 30 to 45 cm depth, and STO in consecutive drought trials. Geo-referencing indicated that the potential drought donors originated in flood-prone locations, corroborating previous hypotheses about the coexistence of flood and drought tolerance within individual *Oryza* genomes. These findings present potential donor accessions, traits, and genomic loci from an AA genome wild relative of rice that, together with the recently developed reference genome, may be useful for further introgression of drought tolerance into the *O. sativa* backgrounds.

## Teaser

Phenotypic and genomic characterization of wild relatives of rice under drought stress reveals promising accessions, showing deeper root growth under drought and lower stomatal density.

## Introduction

Improvement of drought tolerance has been a major breeding challenge for rice (*Oryza sativa*, L.), and in recent decades, the most successful strategy has been to cross drought-tolerant traditional varieties with high-yielding but drought-susceptible modern varieties (Kumar et al. 2014). However, continued improvement of drought tolerance in rice is still needed to meet the challenges posed by climate change and projected demands of a growing population (Lesk et al. 2016; Anderson et al. 2020). One potential source of drought tolerance that has thus far been explored to a lesser extent is from the wild relatives of rice (Menguer et al. 2017). The wild relatives of rice harbor many alleles for both abiotic and biotic stress adaptive traits (Liu et al. 2004; Hajjar and Hodgkin 2007; Brar and Khush 2018) that may be useful for rice breeding programs globally.

The genus *Oryza* consists of 2 domesticated and 25 wild species, harboring a large degree of genetic diversity among them (Vaughan 1994; Sanchez et al. 2013). The *Oryza* species are widely distributed across the globe and vary in their morphology, biology, and genome structure. A number of the wild relatives of rice have been previously identified as sources of useful genes and quantitative trait locus, such as disease and pest resistances, heat- and drought-related traits, and tolerance to toxic elements (Sanchez et al. 2013). Earlier studies have reported that *Oryza longistaminata* and *Oryza rufipogon* possessed improved leaf elongation, leaf membrane stability, and stomatal conductance under drought stress (Liu et al. 2004). Despite this potential, only a small portion of the diversity has been tapped into from the wild relatives of rice and therein lies an unexplored resource from which to screen and identify stress-tolerance traits. Of note, >4,000 wild *Oryza* accessions are maintained between the Rice Genebank of the International Rice Research Institute (IRRI), Philippines, and the National Bio-Resource Project of The National Institute of Genetics (NIG), Japan, and an array of genomic tools has been developed to facilitate their use in rice improvement (Alsantely et al. 2022).

Among the wild relatives of rice, *Oryza glumaepatula* is the only diploid AA genome (i.e. the primary gene pool for cultivated rice) endemic to the new world and is found in South and Central America and the Caribbean (Khush 1997; Vaughan et al. 2003). Depending on its geographical location of origin, the growth habit of *O. glumaepatula* can be annual, biennial, or perennial (Akimoto et al. 1998). The habitat of *O. glumaepatula* is typically in flooded areas, marshes, and river beds (Fuchs et al. 2016). Earlier studies on *O. glumaepatula* have identified genetic regions responsible for erect panicles, late heading (Sanchez et al. 2003), and yield-related traits (Brondani et al. 2002). Physiological studies have characterized the *O. glumaepatula* accessions to have very low stomatal density (STO) but higher photosynthetic parameters compared with *O. sativa* (Kondamudi et al. 2016). These findings suggest that *O. glumaepatula* possesses important morphological, physiological, and agronomic traits that could be used for crop improvement. However, there is very limited information on traits in *O. glumaepatula*, which are related to abiotic stresses such as drought and heat responses that could possibly help improve existing rice varieties.

There is substantial eco-geographic variation exhibited among the AA genome *Oryza* species, and a high degree of adaptive differences among individual accessions within a species are expected (Vaughan et al. 2003). In this study, we identified *O. glumaepatula* as a potential source of drought tolerance based primarily on root traits observed under drought stress and hypothesized that physiological traits from *O. glumaepatula* that confer drought tolerance could be identified. To comprehensively assess the genetic diversity of *O. glumaepatula*, we first assembled a high-quality reference genome using the PacBio SMRT long-read sequencing technology followed by the generation of a high-density single nucleotide polymorphism (SNP) dataset for a diversity panel of 69 *O. glumaepatula* accessions. We then evaluated this panel (Supplemental Table S1) to pinpoint potential sources of drought tolerance in terms of root growth, STO, and maintenance of shoot biomass and to identify the related genetic regions that could be used in breeding programs.

## Results

### *O. glumaepatula* stood out for drought response among 22 *Oryza* species

**Figure 1.**
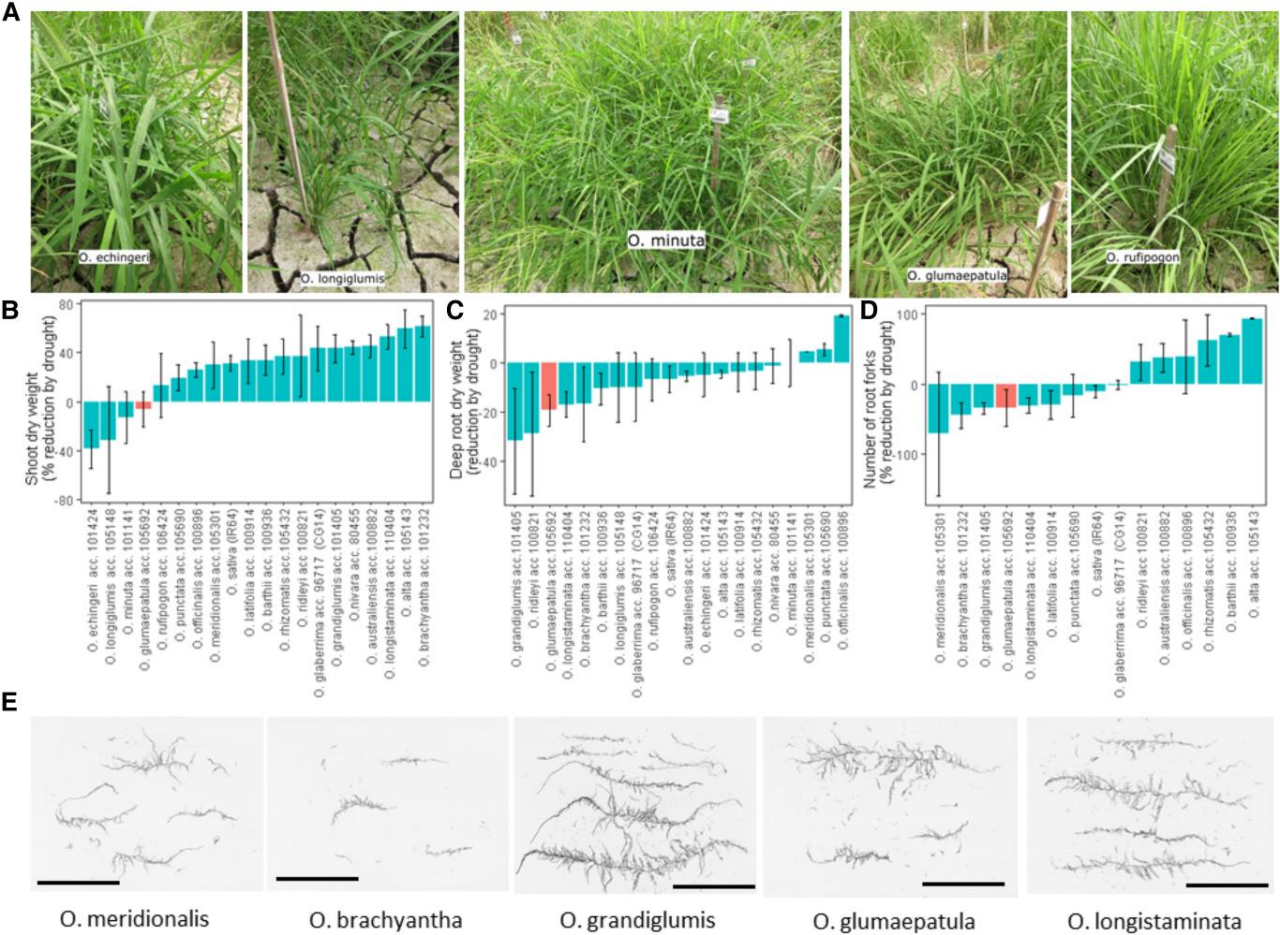
Response of 24 *Oryza* species to drought in greenhouse cylinder (*n* = 4) and screenhouse paddy (*n* = 3) experiments. **A)** The species that were ranked as the top 5 for maintenance of shoot biomass in the drought stress treatment when compared with the WW treatment. Graphs showing the reduction of **B)** shoot biomass (screenhouse), **C)** deep (>30 cm) root dry weight (screenhouse), and **D)** number of root forks (greenhouse) in the drought stress treatment when compared with the WW treatment. *O. glumaepatula* ranked in the top 5 species for maintaining shoot biomass under drought in both experiments. Values shown are means ± Se. **E)** Root images of the species with the highest increase in the number of root forks under drought. The root system from one whole plant in the greenhouse experiment is shown in each image. The size bars indicate a length of 10 cm.

To obtain a preliminary assessment of how the wild relatives of rice respond to drought, we began by screening one accession from each of 22 *Oryza* species (out of 24 species that were planted; Supplemental Table S2) under drought stress and well-watered (WW) conditions in both greenhouse cylinder and screenhouse paddy experiments. *O. glumaepatula* was the only species that ranked among the top 5 species of both experiments for maintaining its shoot dry weight (SDW) under drought when compared with WW conditions (Fig. 1). *O. glumaepatula* also ranked among the top 5 species for maintaining its number of crown roots and number of forks per nodal root in the greenhouse cylinder experiment and for maintaining its deep root length percentage, deep root mass percentage, and leaf osmotic potential (LOP) in the screenhouse paddy experiments under drought when compared with WW conditions (Fig. 1, Supplemental Figs. S1 and S2).

### Drought effects on physiological traits across the *O. glumaepatula* panel

Based on the performance of this representative *O. glumaepatula* accession in comparison with other wild *Oryza* species, we explored the physiological diversity in drought response of a diversity panel of 69 *O. glumaepatula* accessions that originated from the new world (Supplemental Table S3) over 2 growing seasons. In both seasons, drought stress had a significant effect on most of the traits measured (Supplemental Table S4), especially in terms of SDW, plant height (PHT), tiller number (TLN), root dry weight (RDW) at the shallow depth (0 to 15 cm), and crown root number (CRN) (Supplemental Figs. S3 to S5).

**Figure 2.**
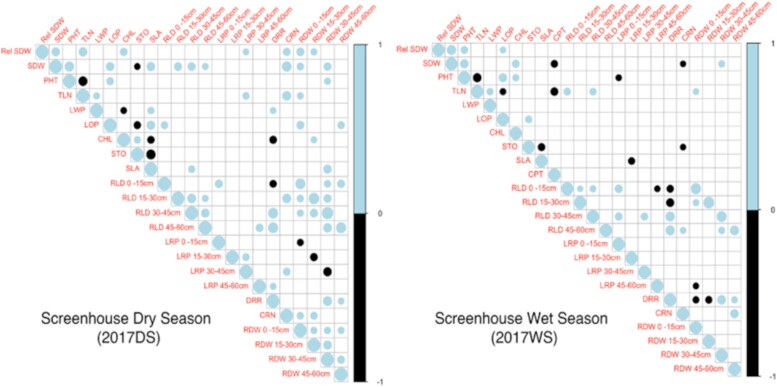
Phenotypic correlation matrices using Pearson correlation coefficient between maintenance of SDW and multiple phenotypic traits under drought stress conditions in both the seasons in which the 69 *O. glumaepatula* accessions were evaluated. Only significant correlation coefficients with *P*-values <0.05 are shown. The size of the dot indicates the magnitude, and the color indicates the direction of correlation (positive or negative) between traits. Rel SDW, relative shoot dry weight; SDW, shoot dry weight; PHT, plant height; TLN, tiller number; LWP, leaf water potential; LOP, leaf osmotic potential; CHL, chlorophyll content; STO, stomatal density; SLA, specific leaf area; RLD, root length density at 0 to 15, 15 to 30, 30 to 45, and 45 to 60 cm depths; LRP, lateral root percentage at 0 to 15, 15 to 30, 30 to 45, and 45 to 60 cm depths; DRR, deep root ratio; CRN, crown root number; RDW, root dry weight at 0 to 15, 15 to 30, 30 to 45, and 45 to 60 cm depths.

Using maintenance of SDW (under drought stress compared with WW conditions) as the direct measure of drought tolerance, different traits were directly correlated with drought tolerance in the 2 seasons studied (Fig. 2). Positive correlations with maintenance of SDW were observed with TLN (*r* = 0.45, *P* < 0.001), chlorophyll content (*r* = 0.36, *P* = 0.003), RDW at shallow depths 0 to 15 cm (*r* = 0.39, *P* < 0.001) and 15 to 30 cm (*r* = 0.27, *P* = 0.026), and CRN (*r* = 0.35, *P* = 0.003) in the drought stress condition during the dry season (Fig. 2). During the wet season, maintenance of SDW was positively correlated with PHT (*r* = 0.36, *P* = 0.003) and LOP (*r* = 0.27, *P* = 0.028) under drought. In both seasons, STO was negatively correlated with specific leaf area under drought stress (dry season, *r* = −0.52, *P* < 0.001; wet season, *r* = −0.38, *P* = 0.002; Fig. 2). PHT and TLN were negatively correlated in both the dry (*r* = −0.48, *P* < 0.001) and wet (*r* = −0.49, *P* < 0.001) seasons under drought stress (Fig. 2).

When maintenance of SDW was regressed with multiple traits measured under drought stress using stepwise multiple regression, PHT, TLN, chlorophyll content, lateral root percentage (LRP) at 30 to 45 cm depth, and RDW at 0 to 15 cm depth were the significant traits that predicted the maintenance of SDW in the dry season (*r*^2^ = 0.37; *P* < 0.001, Table 1). PHT, TLN, CRN, and RDW at 30 to 45 cm depth were the significant traits predicting maintenance of SDW in the wet season (*r*^2^ = 0.48; *P* < 0.001, Table 1).

**Table 1.** Multiple regression of maintenance of shoot dry weight with other phenotypic traits under drought stress in the dry and wet seasons in which the *O. glumaepatula* panel was evaluated

Traits	*T*-value	*P*-value
Dry season^a^		
Plant height	2.29	0.03
Tiller number	3.16	<0.001
Chlorophyll conc. index	2.61	0.01
Lateral root percentage	1.52	0.14
Root dry weight 0 to 15 cm	2.33	0.02
Wet season^b^		
Plant height	4.68	<0.001
Tiller number	4.77	<0.001
Crown root number	−2.08	0.04
Root dry weight 30 to 45 cm	2.09	0.04

The traits from the final stepwise regression model are reported.

^a^Dry season model—multiple *R*^2^: 0.4148, adjusted *R*^2^: 0.3652.

*F*-statistic: 8.363 on 5 and 59 DF, *P*-value: 5.02e−06.

^b^Wet season model—multiple *R*^2^: 0.5169, adjusted *R*^2^: 0.4767.

*F*-statistic: 12.84 on 4 and 48 DF, *P*-value: 3.497e−07.

### Potential *O. glumaepatula* drought donors and their in-depth trait evaluations

**Figure 3.**
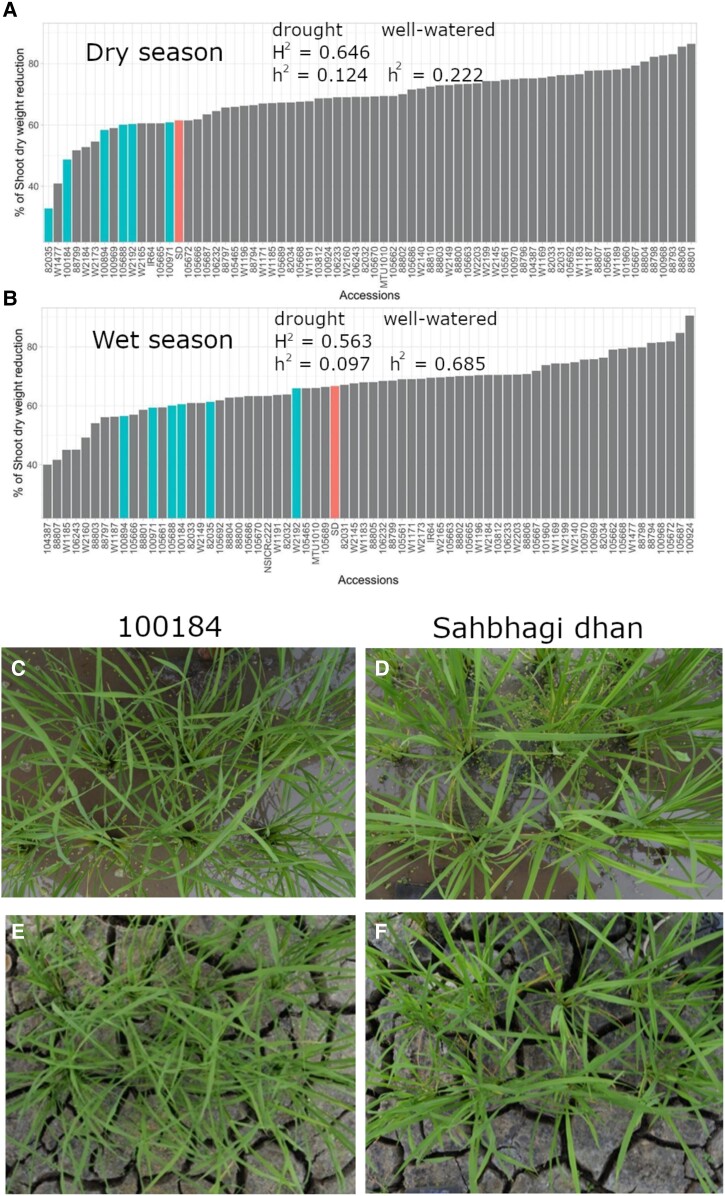
Rank order plot of the proportion of shoot dry weight reduction due to drought stress for all the 69 *O. glumaepatula* accessions along with the *O. sativa* checks in the dry **A)** and wet **B)** seasons (*n* = 3). The potential drought donors and the drought tolerant check variety Sahbhagi dhan (SD) are highlighted. Images shown represent example shoots under WW **C and D)** and drought stress treatment **E and F)** of *O. glumaepatula* accession 100184 and drought tolerant check variety Sahbhagi dhan, respectively. Any nonzero broad sense (H^2^, based on phenotypic data) and narrow sense (h^2^, based on genomic data) heritability values for shoot dry weight in each treatment are indicated.

All the *O. glumaepatula* accessions were ranked from the highest to lowest reduction in SDW along with *O. sativa* checks over both seasons (Fig. 3). Variation in SDW reduction among the *O. glumaepatula* accessions (30% to 85% in the dry season and 40% to 90% in the wet season) was generally smaller than the degree of variation that had been observed across species (−27% to 95% in the greenhouse cylinder experiment [Supplemental Fig. S1] and −38% to 61% in the screenhouse paddy experiment [Supplemental Fig. S2]). Compared with the drought tolerant check variety, Sahbhagi dhan, 6 *O. glumaepatula* accessions (W2192, 82035, 100184, 100894, 100971, and 105688) showed lower SDW reduction in both seasons and were therefore identified as potential drought tolerance donors for use in breeding. We then examined the grouping of those potential donors with respect to Sahbhagi dhan for other physiological traits, of which STO and deep root growth under drought showed the largest degree of advantage for the potential donors.

**Figure 4.**
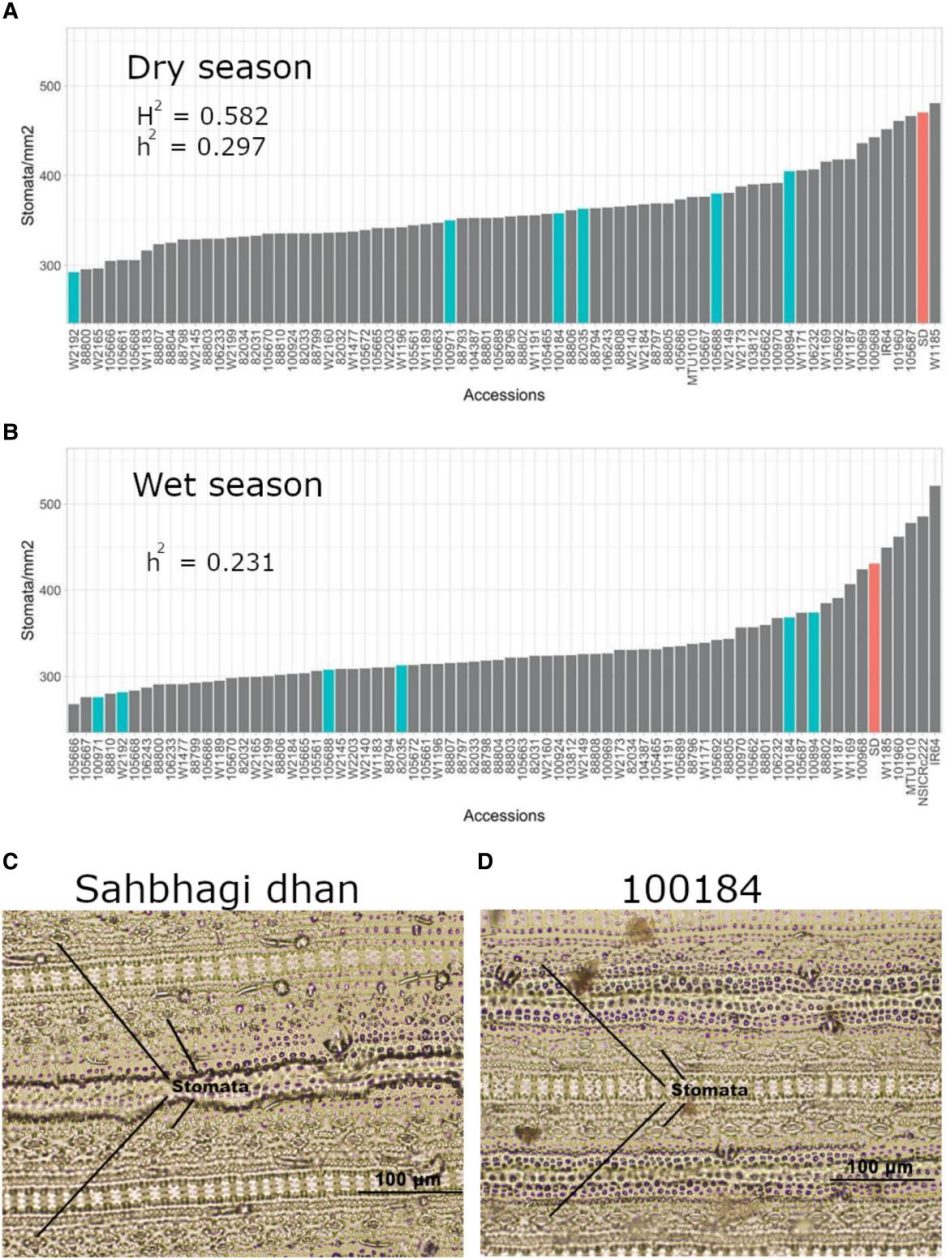
Rank order plot of the stomatal density values under drought conditions for all the 69 *O. glumaepatula* accessions along with the *O. sativa* checks in the dry **A)** and wet **B)** seasons (*n* = 3). The potential drought donors and the drought tolerant check variety Sahbhagi dhan are highlighted. Images shown represent examples of the stomatal density from epidermal peels of the adaxial surface of the youngest fully formed leaf at 20× magnification of **C)** drought tolerant check variety Sahbhagi dhan and **D)***O. glumaepatula* accession 100184. Any nonzero broad sense (H^2^, based on phenotypic data) and narrow sense (h^2^, based on genomic data) heritability values for stomatal density in the drought stress treatment are indicated.

Across entries (the *O. glumaepatula* panel and several *O. sativa* checks), STO under drought stress ranged between 250 and 550 stomata mm^−2^ in both seasons (Fig. 4). Overall, the majority of the *O. glumaepatula* accessions had lower STO than the *O. sativa* checks. The 6 potential donors selected from this experiment all had lower STO than Sahbhagi dhan in both seasons. Accession W2192 appeared to be the most consistent in showing low STO, ranking lowest in the dry season and the 5th lowest in the wet season. Accessions W1185, 101960, 100968, and W1187 had relatively higher STO values than W2192 in both seasons, but these values were still lower than that of the most *O. sativa* accessions evaluated.

**Figure 5.**
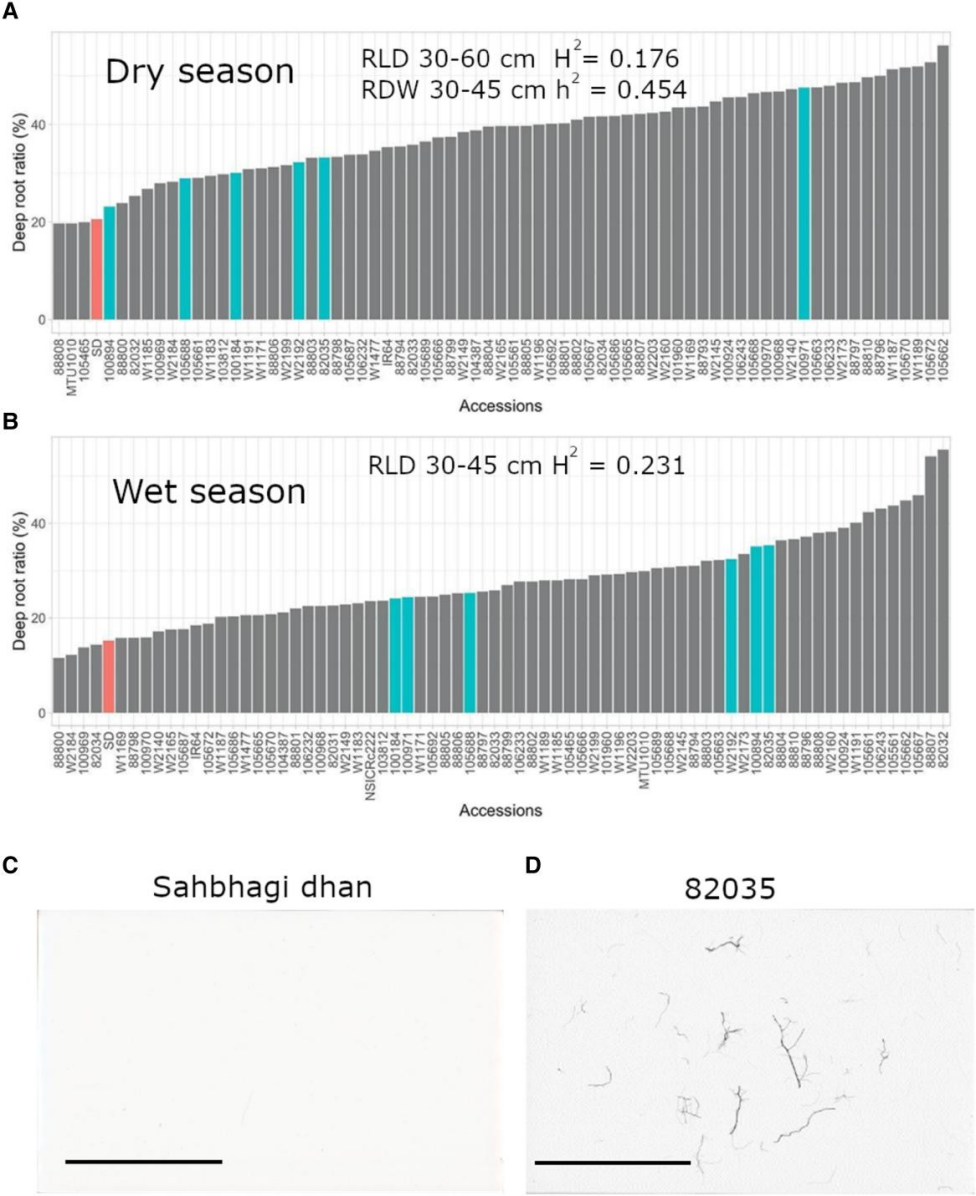
Rank order plot of the proportion of deep roots under drought across all 69 *O. glumaepatula* accessions along with the *O. sativa* checks in the dry **A)** and wet **B)** seasons (*n* = 3). The potential donors and the drought tolerant check variety Sahbhagi dhan are highlighted. Root scan images from a 4-cm diameter soil core at the depth of 45 to 60 cm in the wet season drought stress treatment are shown for **C)** Sahbhagi dhan (no roots detected) and **D)***O. glumaepatula* accession 82035. The absence of roots for the Sahbhagi dhan sample illustrates that the drought tolerant check was not able to form roots at the deepest soil depth sampled, whereas the potential drought donor *O. glumaepatula* acc. 82035 was able to form roots at that depth under drought stress. The size bars indicate a length of 5 cm. Any nonzero broad sense (H^2^, based on phenotypic data) and narrow sense (h^2^, based on genomic data) heritability values are indicated for the relevant root traits (RDW, root dry weight; RLD, root length density) in the drought stress treatments.

The deep root percentage (root length below 30 cm/total root length in the soil core) among the *O. glumaepatula* accessions and *O. sativa* checks ranged between 20% to 56% and 11% to 55% under drought in the dry and wet season experiments, respectively (Fig. 5). All 6 *O. glumaepatula* potential donors identified had higher deep root percentage under drought stress, compared with Sahbhagi dhan in both seasons. However, Sahbhagi dhan had higher deep root percentages than most of the potential donors under WW conditions (Supplemental Fig. S6).

### Sequencing and genetic diversity of the *O. glumaepatula* panel

To develop markers for breeding and begin to understand the underlying molecular mechanisms that control the phenotypic responses to drought described above, we first generated a high-quality genome assembly of a single *O. glumaepatula* (GEN 1233 from the EMBRAPA germplasm collection [BRA 00011525–3], collected in Matrinchã, Goiás, Brazil) using PacBio long-read sequencing and Genome Puzzle Master (GPM) editing (see Materials and Methods). The resulting chromosome level assembly is composed of 33 contigs (N50 = 18.2 Mb) assigned to 12 chromosomes with a total length of 386 Mb and was used as a reference genome sequence to perform all the remaining downstream analyses.

To assess the genetic diversity of the *O. glumaepatula* diversity panel, each accession was Illumina resequenced to a minimum coverage of 3× (Supplemental Table S5). The resequencing data were then mapped to the *O. glumaepatula* RefSeq to call SNP using the genome analysis toolkit (GATK) pipeline (McKenna et al. 2010; see Materials and Methods). SNP calling and subsequent quality filtering (see Materials and Methods) resulted in a dataset of 14,238,207 biallelic SNPs, of which 14,222,318 are on chromosomes 1 to 12 and 15,889 are on unaligned contigs.

Following variant calling, we explored the population structure of the panel as well as associations between the genome and phenotypes. Five samples were found to be genetic outliers, based on significantly lower (*P* < 0.01, *t*-test) transition-to-transversion (*T_s_*/*T_v_*) ratios (2.11 to 2.29, while the rest of the panel had 2.43 to 2.57), indicating possible contamination. Consistent with this hypothesis, 4 of these had a markedly higher number of variant calls compared with the bulk of the panel, as well as elevated number of missing calls (>30%; Supplemental Fig. S8C). The remaining one sample (100894, geographical annotation: Cuttack) was found to contain a higher number of heterozygous SNPs. Three more samples within the bulk of the panel had a higher proportion of heterozygous SNP calls as well.

**Figure 6.**
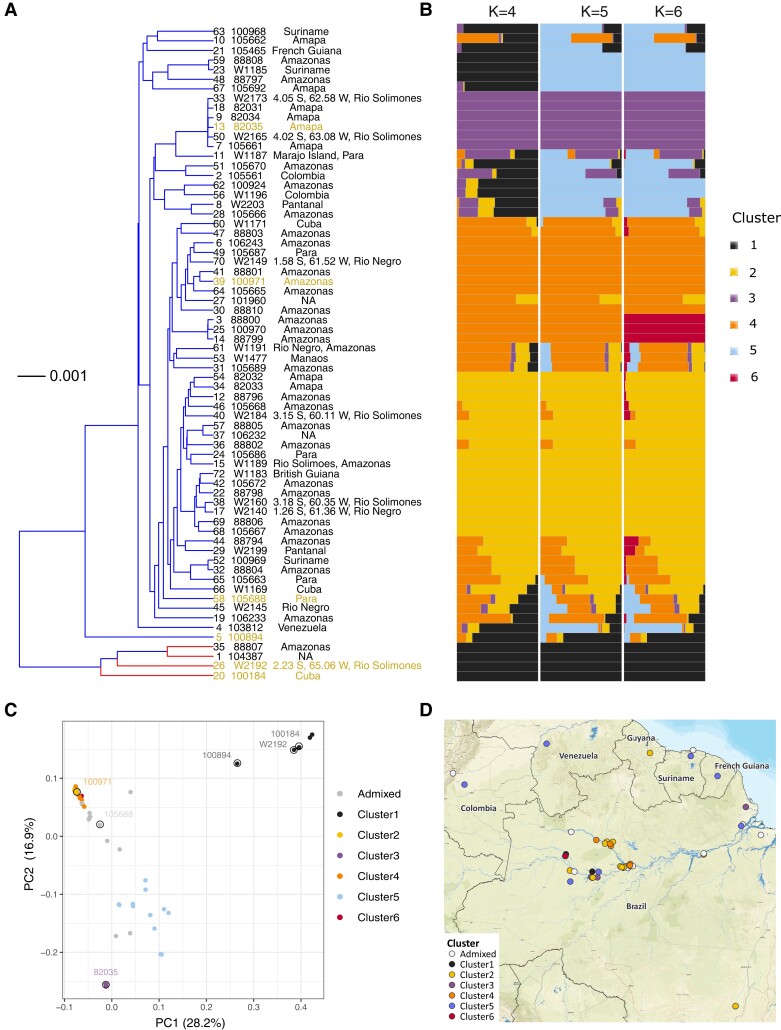
Genetic clustering and geographic distribution of the 69 *O. glumaepatula* accessions in this study. **A)** Neighbor-joining tree of the 69 *O. glumaepatula* accessions using whole-genome sequence data. The names of the 6 potential donors are highlighted. The lines indicating accessions with a high proportion of *O. sativa* genome are highlighted. Text indicates the VCF ID, accession name, and location of origin. The scale bar indicates 0.1% nucleotide sequence divergence. **B)** Population structure analysis generated by ADMIXTURE software. The *O. glumaepatula* accessions are shown on the *y*-axis in the same order as in **A**). The 3 *K* levels shown represent different numbers of ancestral populations considered. Bars indicate the proportion of each genome representing each ancestral population (cluster). **C)** PCA (PC1 to 2) on the whole genome sequence data according to clusters in **B**). An accession was determined to be admixed if none of the clusters exceeded 70% of the genome. **D)** Geographical distribution of accessions based on the passport information. Each point shows one accession, and the cluster representing the maximum proportion of the genome is indicated.

Using a neighbor-joining tree, the 6 potential donor accessions were distributed across several clusters (Fig. 6A). Based on the ADMIXTURE bar plot, using different numbers of ancestral populations to indicate population structure (Fig. 6B), *K* = 6 represented the lowest cross-validation error (Supplemental Fig. S7), and *K* = 4 and *K* = 5 revealed the possible genetic proximity of Clusters 1 and 5 as they merged. Among accessions, 2 potential donors (100184 and W2192) and 2 other accessions (104387 and 88807) had the highest proportion of their genome represented by Cluster 1. Further investigation based on mapping short read sequences of the *O. glumaepatula* accessions along with 2 *O. sativa* varieties to the Nipponbare reference, as well as differences in *T_s_*/*T_v_* ratios, suggests that those 4 accessions share a high proportion of their genome with *O. sativa* (Supplemental Fig. S8, A to D). Phenotypic observation of the seeds (Supplemental Fig. S8E) suggested an *O. glumaepatula* origin of the Cluster 1 accessions based on the dark brown color of the hulls when compared with the lighter color that is typical of *O. sativa* (except accession 100894 whose seeds were lighter-colored). The first axes of a principal component analysis (PCA) on the whole-genome sequence data (Supplemental Fig. S9, top row) were heavily influenced by the introgressed samples (Cluster 1, black), which was consistent with the ADMIXTURE-based clustering. PCA also indicated the 4 Cluster 1 accessions to be well-separated by PC1 (Fig. 6C). The subsequent axes reflect population differentiation as found in the ADMIXTURE analysis (PC3 separates Cluster 3 from Cluster 5 and PC4 separates Cluster 2 from Cluster 4). As indicated by PC3 to 4 (Supplemental Fig. S9), Cluster 3 may share more alleles with Cluster 1 than other clusters, suggesting that the population could have been crossed with *O. sativa* to give rise to Cluster 1. These clusters were well separated by the PCs with and without different levels of minor-frequency allele filtering (Supplemental Fig. S9), indicating that the analysis is detecting real genetic structure.

The geographic distribution of collection sites among accessions within a cluster was varied (Fig. 6D). Representatives of all clusters were found in Brazil. All of the potential donors were collected from within the Amazon River Basin. Cluster 5 had the broadest geographic dispersal. Clusters 2 and 4 were found in similar locations.

Based on the consistent ranking of the *O. glumaepatula* accessions with the least reduction in shoot biomass under drought (i.e. the potential donors) as showing lower STO and deep root ratio (DRR) compared with the drought tolerant check Sahbhagi dhan, we examined the genomic regions associated with those traits (with the introgressed accessions excluded). Between the 2 seasons of study, no significant GWAS peaks were detected for relative SDW, but three 100-kb windows were identified for SDW under drought (Table 2, Supplemental Fig. S10). Six significant SNPs colocated in the 2 seasons for STO under drought stress (Table 2, Supplemental Fig. S10). Although the GWAS model did not converge for the DRR data of the drought stress treatment, four 100-kb windows colocated between seasons for root length density (RLD) at the 30 to 45 cm depth under drought stress (Table 2, Supplemental Fig. S10). These results should be considered as preliminary given the relatively small set of genotypes used for GWAS in this study.

**Table 2.** Genomic loci associated with drought-response traits characteristic of the potential *O. glumaepatula* donors that colocated between seasons: (**A)** colocating 100-kb windows for SDW significant SNPs, with a threshold of −log_10_(*P*) > 3.5. (**B**) SNPs colocating for stomatal density by MLM, filtered, where at least one season has −log_10_(*P*) > 5 (and the other >4), (**C)** colocating 100-kb windows for RLD at the 30 to 45 cm soil depth by MLM with 2 principal components, with a threshold of −log_10_(*P*) > 3.0

(A) Shoot dry weight				
Chr	100-kb window start (Mb)		SDW dry season drought stress	SDW wet season drought stress
			−Log_10_*P*	−Log_10_*P*
1	43.1		3.66145	3.84213
3	1.4		3.95904	4.76544
6	32.3		4.54859	5.41999
(B) Stomatal density				
Chr	SNP ID	Position (bp)	Stomatal density dry season drought stress	Stomatal density wet season drought stress
			−Log_10_*P*	−Log_10_*P*
3	3_20764471_G_A	20764471	5.115006	5.518272
3	3_7967736_A_G	7967736	6.562149	7.071129
3	3_7968821_G_A	7968821	5.367097	7.550906
3	3_7992024_C_T	7992024	5.572183	7.669839
5	5_25639897_T_C	25639897	5.010085	5.081893
10	10_14029110_C_T	14029110	5.138574	5.446336
(C) Root length density at 30 to 45 cm depth				
Chr	100-kb window start (Mb)		RLD_30 to 45 cm dry season drought stress	RLD_30 to 45 cm wet season drought stress
			−Log_10_*P*	−Log_10_*P*
2	12.3		3.44939	4.78743
5	11.1		3.34197	4.17064
6	26		3.23524	4.20333
5	7.7		3.04967	4.9519

## Discussion

The large distribution among accessions for the traits measured in this study reveals the variation in drought response within *O. glumaepatula* and suggests that different combinations of traits can contribute to drought tolerance in different accessions. Given the large variation in phenology and biomass, we used maintenance of shoot biomass as our standard measure of drought tolerance in this study and selected 6 *O. glumaepatula* accessions with the greatest degree of maintenance of shoot biomass under drought. Some of those potential donors showed interesting admixtures—likely with *O. sativa*—that may help explain their adaptability to drought stress conditions.

The trend of the 6 *O. glumaepatula* accessions showing higher deep root percentages than Sahbhagi dhan under drought stress (Fig. 5), but lower deep root percentages under WW conditions (Supplemental Fig. S6), suggests that *O. glumaepatula* could be a potential source for drought avoidance mechanisms via plasticity in deep root growth. A hypothesized advantage of plasticity in deep root growth over constitutive deep root growth is that the plant does not invest resources unnecessarily when deep root growth is not needed, e.g. under WW conditions, which could help maintain higher grain yields (Schneider and Lynch 2020). We also observed that maintenance of SDW was positively correlated with shallow RDW in the dry season but not in the wet season, which may be due to the differences in soil drying rates between the 2 seasons. These results further suggest a relatively high level of plasticity in *O. glumaepatula* root growth in response to different types of drought.

One trait for which all *O. glumaepatula* accessions stood out was STO. Overall, most of the *O. glumaepatula* accessions showed lower STO when compared with the *O. sativa* checks in this study. Low STO is a trait hypothesized to improve crop water use efficiency under drought and is therefore a key candidate for trait-based breeding, but its potential tradeoffs for yield potential must be assessed. *O. glumaepatula* accession 104387 was previously identified among wild relatives of rice for its very low STO despite showing high photosynthesis rates (Kondamudi et al. 2016). Caine et al. (2019) reported a lack of reduction in grain yield in rice lines edited to overexpress OsEPF1 with reduced STO despite their lower stomatal conductance and photosynthesis rates. Those studies indicate that lower STO may not necessarily result in tradeoffs in terms of grain yield under WW conditions. In addition, recently developed drought breeding lines have shown slightly lower STO than the check variety IR64 across different environments (Kumar et al. 2021), further highlighting this trait as beneficial for yield and suggesting that *O. glumaepatula* lines with even lower STO than Sahbhagi dhan could be promising drought donors for breeding. The consistent GWAS peaks identified for STO in this study can be used in developing markers that could be used for introgressing low STO into an *O. sativa* background.

The lack of a significant relationship between canopy temperature (CPT) and STO in this study may reflect the interaction between reduced STO (which is likely to raise CPT by reducing evaporative cooling) and greater maintenance of shoot biomass (which is likely to reduce the CPT measurement via higher groundcover). STO was negatively correlated with specific leaf area in both seasons under drought, indicating that leaves with higher STO tended to be thicker, which is in agreement with observations in *Leymus chinensis*, another C_3_ grass species (Xu and Zhou 2008). Furthermore, while phenotyping for STO in this study, it was observed that the leaf imprint quality of *O. glumaepatula* accessions was distinctly clearer than that of the *O. sativa* checks. Two possible reasons could be the variation in the leaf surface structure or physiological age of the leaf as the *O. sativa* checks flower earlier. Therefore, further investigation of *O. glumaepatula* leaf structure may provide additional insights into potential drought tolerance traits that could be conferred by *O. glumaepatula*. This observation also has implications for potential high-throughput screening of *O. glumaepatula* accessions that are currently not possible for *O. sativa* due to its complex leaf surface structure.

The 6 selected *O. glumaepatula* accessions that had higher maintenance of SDW all showed both lower STO and higher deep root percentage than the drought-tolerant *O. sativa* check Sahbhagi dhan under drought stress conditions (Figs. 4 and 5). Earlier studies in *O. glumaepatula* have been focused on agronomic traits and used to improve the yield of *O. sativa* varieties in South America (e.g. Brondani et al. 2002; Rangel et al. 2013). Given the range of traits related to the performance of our 6 selected *O. glumaepatula* accessions as potential drought donors as well as their well-distributed relatedness within the species, a number of combinations of these promising characteristics of *O. glumaepatula* could be further evaluated to potentially be used in breeding rice for drought conditions.

It was notable that the *O. glumaepatula* potential drought donors originated near flood-prone regions of the Amazon River Basin. Previous work has also reported some beneficial drought traits coming from Riz Africain de Mali *Oryza glaberrima* lines collected from flood-prone areas (Ndjiondjop et al. 2010) and their progeny from crosses with *O. sativa* (Kijoji et al. 2013) and from deepwater rice based on deep root growth and low CPT (Rayada; Henry et al. 2011) and based on gene expression under drought (Bhadoia: Groen et al. 2022). The coexistence of drought and flooding tolerance in rice has been hypothesized to be related to ethylene-responsive pathways being common to both drought and flood response (Bin Rahman and Zhang 2016; Bin Rahman and Zhang 2022). These common pathways may confer a certain degree of plasticity in response to both types of stress.

Another notable aspect of origin of the *O. glumaepatula* accessions having been collected along riverways (Fig. 6D) is regarding the geographic distribution of the accessions, especially since there was no apparent geographic grouping of population structure clusters. Riverways may have facilitated human dispersal of *O. glumaepatula* accessions prior to collection either through transportation of seed by boat or through river dragging that has been documented to disperse *O. glumaepatula* (de Campos Vaz et al. 2009), and it may be that riverways were used by the germplasm collectors to acquire the seed. Furthermore, *O. glumaepatula* has a habit of stems breaking and floating down the river and becoming reestablished at other locations (Akimoto et al. 1998; Abreu et al. 2015). On the other hand, the distribution of the potential drought donors among several clusters (Fig. 6C) suggests that adaptive alleles are present in different genetic groups, independent of geographic distribution. Whether these adaptive alleles represent shared ancestral alleles that entered the populations through sorting or introgression, or from novel variation that arose independently in different groups, is an interesting question that needs further investigation. Furthermore, the similar drought tolerance traits observed across those 6 potential donors implies that a few genomic regions that are beneficial for these traits might appear in several subpopulations. Another possible explanation for the distribution of drought tolerance in the panel among geographic locations and population structure clusters is introgression: the 4 introgressed accessions may represent some crossing events that occurred before collection and deposition in Genebanks. Likewise, the similarities between Cluster 1 and Cluster 5 (Fig. 6B) may represent possible crosses between *O. glumaepatula* and *O. sativa* most likely through natural gene flow (Karasawa et al. 2007); these lines may also contain traces of introgressions from other *Oryza* species, as proposed by Stein et al. (2018) between *Oryza barthii* and *O. sativa.* Introgression into wild relatives of rice has been reported previously (Brondani et al. 2005; Fuchs et al. 2016) and has been cited as both a key evolutionary mechanism for adaptation as well as a major concern for conservation of wild populations in nature. In the case of the potential drought donors identified here, the high degree of *O. sativa* introgression may indicate that the related drought-response genes are well-adapted to the *O. sativa* background, which would be promising for their use in breeding.

## Conclusions

Although some progress has been made in improving the level of drought tolerance of cultivated rice (*O. sativa*), this has mainly been through introgressions from the aus and indica subgroups, and these sources of drought tolerance are becoming exhausted in terms of their utility in rice improvement. More and diverse introgressions are needed to further improve the productivity of rice in the face of climate change, specifically under the increased incidence of drought globally. The potential drought donors and genomic regions associated with their increased root length at depth and lower STO identified here can be crossed with improved *O. sativa* backgrounds, which is feasible given the AA genome of *O. glumaepatula*. The sequence data of the 69 *O. glumaepatula* accessions mapped to the high-quality reference genome presented here will be beneficial for allele mining and dissecting the genetic basis of drought response traits. With these phenotypic and genetic resources from *O. glumaepatula*, researchers can advance the use of wild relatives of rice for developing new *O. sativa* varieties that are better adapted to climate change.

## Materials and methods

### Plant material

Two sets of experiments were conducted as part of this study to screen for root growth and drought response among wild relatives of rice: (i) evaluation of 24 *Oryza* species, and (ii) evaluation of an *O. glumaepatula* diversity panel.

### Sequencing and assembly of *O. glumaepatula* reference genome

High molecular weight DNA was extracted from young leaves adopting the protocol of Russell and Sambrook (2001) with minor modifications. PacBio library preparation followed the 20 kb protocol (https://www.pacb.com/wp-content/uploads/2015/09/Insert-SMRTbell-Template-Prep-Kit-1.0.pdf) and was sequenced on a PacBio Sequel sequencer with movie collection time of 10 h. The raw sequence data were assembled with FALCON (2017.06.28–18.01-py2.7-ucs4) (Chin et al. 2016), Canu (v1.5) (Koren et al. 2017), and MECAT (v1.3) (Xiao et al. 2017), respectively. Detailed statistics of each assembly are shown in Supplemental Table S6. Canu de novo assembled contigs were used as backbone to build pseudomolecules using GPM Zhang et al. (2016), and then all pseudomolecule components were polished twice with PacBio raw reads using Arrow (https://github.com/PacificBiosciences/gcpp). During manual GPM editing process, MH63RS2 (NCBI Accession# GCA_001623365.2) genome sequences were used as guiding sequences to order and orientate the Canu-assembled contigs; if any contigs in FALCON or MECAT assemblies could bridge a gap between 2 Canu-assembled contigs, they would be integrated to increase the contiguity of the final assembly. The statistics of OgluRS3 sequences are also in Supplemental Table S6. The final assembly was submitted to the NCBI GenBank under the WGS accession ALNU03000000, and the genome is available at Gramene_Oryza https://oryza-ensembl.gramene.org/Oryza_glumaepatula/Info/Index.

The genome assembly (ALNU00000000.3) was assessed by BUSCO analysis (Manni et al. 2021) based on core genes of Viridiplantae, which show the 99.0% completeness for the genome. Compared with the previous *O. glumaepatula*_v1.5 assembly (containing 17,900 gaps) and the MH63RS3 gap-free genome, our latest assembly (containing only 21 gaps in chromosomes) showed high collinearity and completeness (Supplemental Fig. S11). The gene annotation data are available at Gramene: https://ftp.gramene.org/oryza/release-6/gff3/oryza_glumaepatula/oryza_glumaepatula_core_3_87_4.gff.

### Evaluation of *Oryza* species

One accession from each of 24 *Oryza* species was selected based on their inclusion in the *Oryza* Map Alignment Project (Wing et al. 2005) and obtained from maintained plant stocks at the IRRI, Los Baños, Philippines. The *Oryza* species were grown in one greenhouse cylinder experiment and one screenhouse paddy experiment (Supplemental Figs. S1 and S2).

The greenhouse cylinder experiment was conducted from March to April 2015. The cylinders were comprised of soil-filled tubes (5 cm diam, 40 cm height, 1.14 g cm^−3^ bulk density) including an inner mylar tube liner with a freely draining fabric bottom and an outer PVC cylinder with a sealed bottom. Plants were germinated from seed, except for *O. rufipogon*, *O. australiensis*, *O. schlechteri*, and *O. coarctata*, which were grown from clones: newly emerged shoots that were separated from the main plant of each wild species. All seedlings (either from seed or from clones) were established in soil-filled seedling trays before transplanting to the greenhouse cylinders. Plants were grown under WW (flooded 7 days after transplanting [DAT]) and drought (DD: drydown starting at 75% of field capacity from the time of transplanting the seedlings into the experimental cylinders) treatments in 4 replicates per treatment for 21 d. Of the 24 *Oryza* species planted in the cylinder experiment, only 16 survived for a duration sufficient to collect data (Supplemental Table S2).

Phenotypic screening of the 24 *Oryza* species in a paddy field within a screenhouse was conducted under both WW and drought stressed conditions in the 2016 dry season (January to March 2016) at IRRI, Los Baños, Philippines (Supplemental Fig. S12). Plants were pregerminated on a filter paper before establishing in soil-filled seedling trays, except for *O. longiglumis*, *O. longistaminata*, and *O. meyeriana*, which were germinated in a test tube containing 1/4 MS media, and then placed in a dark room until its germination after 2 to 3 d. The germinated seeds were placed in a lighted room and later transferred to soil-filled trays when the roots were well developed. *O. schlechteri* and *O. coarctata* were clonally propagated due to difficulty to germinate the seeds. Clonal propagation was done by separating a newly sprouted tiller from the base of a mature plant, and each individual tiller was planted in pots filled with soil. Seedlings were then transplanted into the main experimental paddy. The plot size was 2 rows × 6 hills with 0.25 m between rows and 0.2 m between hills, although the actual number of plants per plot ranged from 1 to 12 based on germination and establishment. A one-row border space was left between adjacent plots. In the case of the WW condition, the soil was flooded throughout the growing season, and in the case of the drought-stressed treatment, the field was initially flooded and then drained to impose drought stress during the vegetative stage. The layout was according to a randomized complete block in a split plot design with 3 replications. The drought stress treatment was initiated at 34 DAT, and the experiment was continued until 68 DAT. Of the *Oryza* species planted, 21 survived for a duration sufficient to collect data (Supplemental Table S2).

### Evaluation of *O. glumaepatula* accessions

A diversity panel of 69 accessions of species *O. glumaepatula* was evaluated in 2 screenhouse paddy experiments. Of the 69 accessions, 50 were obtained from the International Rice Genebank Collection (IRRI) and 19 accessions from the National Institute of Genetics, Japan (Supplemental Table S3).

Screenhouse experiments evaluating the panel of *O. glumaepatula* accessions were conducted in the 2017 dry season (February 2017 to May 2017) and wet season (November 2017 to February 2018) at IRRI. The seeds were manually dehulled and germinated in Petri dishes prior to sowing in germination trays to achieve better germination rates, given the limited seed availability and the possibility of low germination rates and seed dormancy typically observed in wild relatives of rice. Two weeks after emergence in the seedling trays, the seedlings were transplanted in the screenhouse in drought and WW treatments as described above. The drought stress treatment was initiated at 37 and 35 DAT in the dry and wet seasons, respectively, and rewatering in the drought stress treatment was done at 74 and 85 in the dry and wet seasons, respectively.

### Environmental characterization

Average temperature/humidity values were 29.3°C/68.3% in the greenhouse cylinder experiment, 26.5°C/81.6% during the screenhouse experiment on *Oryza* species (measured outdoors), and 28.5°C/61% and 26.8°C/58.7% in the dry season and wet season experiments (measured inside the screenhouse) on the *O. glumaepatula* panel, respectively.

Soil moisture levels of the drought stress treatment were monitored in the screenhouse experiments by tensiometers (Soilmoisture Equipment Corp.) installed at a depth of 30 cm in each replicate. Between the 2 *O. glumaepatula* experiments, the soil water potential indicated that the stress was more severe at the vegetative phase (around 50 DAT) during the dry season when the soil water potential at a depth of 30 cm reached −60 kPa, compared with the wet season when the minimum soil water potential reached −10 kPa (Supplemental Fig. S13).

### Phenotypic characterization of drought response traits

Shoots were sampled in all experiments: the greenhouse cylinder experiment on *Oryza* species was harvested at 21 DAT, and destructive shoot sampling in screenhouse experiments was performed on 2 plants per plot at 64 DAT in the *Oryza* species and at 60 to 64 and 60 to 69 DAT (2017DS-2017WS) in the *O. glumaepatula* panel in the WW and drought stress treatments, respectively. PHT and TLN were measured, and the shoots were dried at 70°C to determine the SDWs. Maintenance of shoot biomass was calculated to normalize for the diverse plant size and flowering time among accessions and was compared with all the traits measured under the drought stress treatment. This normalization of SDW was performed to account for the high natural variation in flowering time, photoperiod sensitivity, and plant biomass among the *O. glumaepatula* accessions, which made harvest of grain yield unattainable in many accessions.

Concurrent to the shoot sampling, root crown sampling (whole root sampling in greenhouse cylinder experiment) was conducted. In the greenhouse cylinder experiment, the depth of the longest nodal root was noted before washing the entire root system from the soil. In the screenhouse experiments, root crowns were excavated to a depth of 20 cm, cleaned, and stored in ethanol. Root crowns were manually counted to determine the total CRN of each sampled plant.

Leaf water potential measurements and sampling for LOP were conducted at 64 DAT in the screenhouse experiment on the *Oryza* species and at 50 and 57 DAT (2017DS-2017WS) for both WW and drought stress treatments on the *O. glumaepatula* panel. Two youngest fully expanded leaves from 2 different plants were selected and were immediately inserted into a pressure chamber (3000HGBL Plant Water Status Console, Soilmoisture Equipment Corp.) to record the leaf water potential and then stored in a plastic bag with a small amount of water and placed in an ice chest for further analysis. The same leaf on which leaf water potential was determined was used to determine leaf area (LI-3100C, Li-Cor Inc.). After determining leaf area, the leaves were placed inside coin envelopes and dried at 70°C to determine the leaf dry weight and calculate the specific leaf area (cm^2^ mg^−1^).

For LOP measurements, 3 youngest fully expanded leaves from different plants were harvested from each plot and immediately folded and placed inside a syringe covered with aluminum foil and stored in ice. The syringes were then stored at −20°C. The sap was extracted by squeezing the samples in the syringe, and the LOP was determined using a vapor pressure osmometer (Vapro model 5520, Wescor, Logan, UT, USA).

In the *O. glumaepatula* diversity panel experiments, nondestructive chlorophyll content measurements (CCM-200, Apogee Instruments) were conducted on 2 youngest, healthy, and fully expanded leaves selected from 2 plants in each plot at 3 different positions on a single leaf at 36 to 37 and 65 DAT (2017DS-2017WS) for both WW and drought stress treatments. CPT was measured at 62 DAT (2017WS) in both the WW and drought stress treatments of the *O. glumaepatula* panel experiments on a clear sunny afternoon. A hand-held infrared sensor (Apogee Instruments) was positioned about 0.5 m above the canopy and held at 45° angle during the measurement. Three readings were taken for each plot for all 3 replications in the screenhouse. STO was determined on the *O. glumaepatula* panel experiments for which 2 youngest fully expanded leaves were selected from each plot at 53 to 54 and 58 DAT (2017DS-2017WS) in the WW and at 58 to 59 and 56 DAT (2017DS-2017WS) in the drought stress treatments. Clear nail polish was applied to a 2 to 3 cm length on the middle of the leaf's adaxial surface and allowed to dry for about 5 min. Cellophane sticky tape was placed on the nail polish patch and gentle pressure was applied. The tape was then carefully peeled to remove it along with the intact nail polish imprint (the stomatal peel), which was then mounted on a glass slide and viewed under 20× magnification with the brightfield using a compound microscope (Olympus microscope, BX51). The stomatal number was counted on 3 fields of view for each sample. STO was calculated by dividing the stomatal number by the field of view area which was 0.1452 mm^2^.

### Root sampling

Soil cores were extracted in the screenhouse experiments between 2 plants in a plot for all 3 replications to a depth of 60 cm using a 4 cm diameter core sampler on 68 DAT in the 24 *Oryza* species and at 52 to 53 and 63 to 64 DAT (2017DS-2017WS) in the WW treatment and at 56 to 57 and 70 to 72 DAT (2017DS-2017WS) in the drought stress treatment of the *O. glumaepatula* panel. The cores were then split into 4 depth segments 0 to 15, 15 to 30, 30 to 45, and 45 to 60 cm. These soil core segments were washed thoroughly to remove the soil and other major impurities using a fine sieve, and the roots were stored in 50% ethanol (v/v) at 4°C. The stored root samples were then scanned at 600 dpi (Epson Calibrated Color Optical Scanner STD4800), and the images were analyzed using WinRHIZOPro v. 2013e (Regent Instruments) to determine total root length and also lengths within different diameter classes. RLD for each depth was calculated by dividing the scanned root length by the volume of each soil core segment (188.5 cm^3^). The LRP was calculated by dividing the total root length with diameter <0.2 mm by the total root length in all diameter classes times 100. DRR was calculated using the formula below:


$$\begin{array}{rl} & \mathrm{Deep}\;\mathrm{root}\;\mathrm{ratio}\\ & =\frac{\mathrm{Sum}\;\mathrm{root}\;\mathrm{lengths}\;(30\;\mathrm{to}\;45,\;45\;\mathrm{to}\;60\;\mathrm{cm})}{\mathrm{Sum}\;\mathrm{root}\;\mathrm{lengths}\;(0\;\;\mathrm{to}\;15,\;15\;\mathrm{to}\;30,\;30\;\mathrm{to}\;45,\;45\;\mathrm{to}\;60\;\mathrm{cm})} \end{array}$$

The scanned roots were then stored in an envelope and dried at 70°C to determine the RDW. The RDWs were divided by the volume of each core (188.5 cm^3^) to estimate the RDW density. In the greenhouse cylinder experiment, the whole root system was scanned as described above to determine total root length, number of root forks, LRP, and the RDW was divided by the SDW to calculate root:shoot ratio.

### Statistical analysis

All data analysis was conducted using R v. 3.5.2 (R Core Team 2018). The genotypic mean values for all traits were used to calculate the Pearson's correlation coefficients between the traits, and the correlation plot was constructed using the corrplot package (Wei et al. 2017). Stepwise multiple regression analysis was done with the relative SDW as the dependent variable and all other phenotypic traits under drought stress as independent variables.

### Genotyping and genetic analysis

Whole genome sequencing and variant calling of all 69 *O. glumaepatula* accessions were performed at the University of Arizona, USA, using DNA extracted from leaf tissue from the 2017WS screenhouse experiment. Using BWA-MEM (Li and Durbin 2009) with the default parameters, all clean reads were mapped to the *O. glumaepatula* reference genome. PCR duplicates of reads were removed using the Picard program (http://broadinstitute.github.io/picard), and reads around InDels were realigned using the IndelRealigner option in the GATK (McKenna et al. 2010). GATK was used to identify SNPs using the unique mapping data. SNPs were then applied hard filtering with parameters settings “QUAL< 30.0|| QD< 2.0|| MQ < 40.0,” where QUAL is the overall variant quality, QD is the quality by depth (a normalized quality per number of reads supporting alternate alleles), and MQ is the mapping quality. Of 69 samples, one sample had a very high number of missing calls (accession 105672) and was therefore removed from the dataset for some analyses (PCA). The full SNP dataset was used for phylogenetic tree construction and PCA in Supplemental Fig. S9; prior to running other population structure analyses, the dataset was filtered by requiring minor allele count to be at least 8, number of missing genotypes at most 10, and number of heterozygous calls per SNP at most 8, resulting in a set with 915,349 SNPs. For the ADMIXTURE analysis (Alexander et al. 2009) and kinship matrix construction, we also generated an LD pruned subset using PLINK 1.9 (Chang et al. 2015) using parameters *r*^2^ = 0.85 and window size 50 kb, resulting in a set of 238,751 SNPs.

A model-based population structure analysis implemented in the software ADMIXTURE was run with the number of putative ancestral populations (*K*) ranging from 2 to 9. For each *K* value, we generated 20 randomly thinned SNP subsets of the LD pruned set, by removing every SNP with probability *P* = 0.4 (using PLINK –thin command). We ran ADMIXTURE on each thinned subset and aligned the resulting *Q* matrices using the R package “pophelper” (Francis 2017), which was also used for creating the ADMIXTURE bar plot. Upon removal of aberrant runs, the final *Q*-matrices were averaged and aligned using the same package. The lowest cross-validation error appeared at *K* = 6; thus, it was chosen for defining genetic groups (clusters). The clusters were defined as follows: if the admixture proportion from a single ancestral population exceeded 70%, the sample was assigned to the corresponding cluster, otherwise it was classified as admixed. The R package “phangorn” v.2.7.1 (Schliep 2011) was used for phylogenetic analysis (neighbor-joining tree).

### Genome-wide association study and colocation analysis

Genotype data were further filtered by minor allele count (>8) and genotyping rate (>80%). Genome-wide efficient mixed model analysis (GEMMA) software (Zhou and Stephens 2012) was used to run MLM-based GWAS. For each trait, a null GWAS model (i.e. mixed model with kinship, covariates, but no test SNP) was run, and Bayesian Information Criterion (BIC) value was calculated. For all traits considered, the optimal BIC value was attained at zero covariates; however, the values of BIC were not substantially different when 1 or 2 principal components were added; thus, we also evaluated MLM models with up to 2 principal components as covariates. The 4 introgressed accessions that were identified from the ADMIXTURE analysis were excluded from the GWAS. PCA was done in PLINK v1.9. The kinship matrix was computed in GEMMA using -gk 1 command-line option.

## Supplementary Material

kiad485_Supplementary_Data

## Data Availability

All phenotypic data will be made available on the IRRI Dataverse site upon publication. The *O. glumaepatula* reference genome assembly was submitted to NCBI GenBank under the WGS accession ALNU03000000. The genotype data for the *O. glumaepatula* panel is available on the IRRI Dataverse site at https://dataverse.harvard.edu/dataset.xhtml?persistentId=doi:10.7910/DVN/U40WOU

## Supplemental data

The following materials are available in the online version of this article.

**Supplemental Figure S1.** Screening of 24 *Oryza* species in a greenhouse cylinder study at IRRI during the 2015 dry season.

**Supplemental Figure S2.** Screening of 24 *Oryza* species in a screenhouse paddy study at IRRI during the 2016 dry season.

**Supplemental Figure S3.** Histograms of shoot traits measured on the *O. glumaepatula* panel under well-watered (W) and drought stressed (D) conditions in both seasons.

**Supplemental Figure S4.** Histograms of RLD (cm cm^−3^) and RDW within each depth increment measured on the *O. glumaepatula* panel under well-watered (W) and drought stressed (D) conditions in both seasons.

**Supplemental Figure S5.** Histograms of the percent of total root length as lateral roots within each depth increment and CRN measured on the *O. glumaepatula* panel under well-watered (W) and drought stressed (D) conditions in both seasons.

**Supplemental Figure S6.** Rank order plot of the proportion of deep roots under WW conditions.

**Supplemental Figure S7.** CV error plot for selection of the “best K” for the population structure analysis shown in Fig. 6.

**Supplemental Figure S8.** Dissecting the genetic identity of the 4 Cluster 1 accessions.

**Supplemental Figure S9.** Comparison of PCAs with and without different levels of filtering.

**Supplemental Figure S10.** Manhattan plots and QQ plots for SDW, STO, and RLD at the 30 to 45 cm depth.

**Supplemental Figure S11.** Collinearity of the *O. glumaeptaula* reference genome assembly with the Minghui 63 gap-free assembly MH63RS3.

**Supplemental Figure S12.** Screening of *O. glumaepatula* accessions in the screenhouse facility at IRRI during the 2017 dry season experiment.

**Supplemental Figure S13.** Soil water potential readings from a soil depth for 30 cm using tensiometers in the screenhouse experiments.

**Supplemental Table S1.** List of the experiments conducted in this study.

**Supplemental Table S2.** List of the accessions of *Oryza* species screened for root growth and drought response in greenhouse cylinder and screenhouse paddy experiments.

**Supplemental Table S3.** Details of *O. glumaepatula* accessions evaluated in this study.

**Supplemental Table S4.** *O. glumaepatula* panel: ANOVA results of multiple phenotypic traits measured in dry and wet seasons for the *O. glumaepatula* accessions.

**Supplemental Table S5.** Summary of data for each *O. glumaepatula* accession in this study by Illumina resequencing to a coverage of 3×: sequencing depth, and proportion of reads mapped.

**Supplemental Table S6.** Statistics of different assemblies before and after GPM editing.

## Dive Curated Terms

The following phenotypic, genotypic, and functional terms are of significance to the work described in this paper:
