# Supplementary material for: Oryza glumaepatula: A wild relative to improve drought tolerance in cultivated rice
Source: Plant Physiol. 2023 Sep 4;193(4):2381–97. doi: 10.1093/plphys/kiad485 (PMC10663109; doi:10.1093/plphys/kiad485)
Supplement: kiad485_Supplementary_Data [file kiad485_supplementary_data.pdf]

Thathapalli Prakash et al

In pursuit of wild relatives of rice to improve drought tolerance in cultivated rice: *Oryza glumaepatula*

Supplemental material

Supplemental Table S1. List of the experiments conducted in this study. DS: dry season, WS: wet season, RCBD: randomized complete block design.

Year	Experiment	Material	Genotypes	Treatments	Experimental design	Reps/ blocks
2015	Greenhouse cylinder study	Wild *Oryza* species	24	Well-watered, drought	RCBD	4
2016	Screenhouse dry season (2016DS)	Wild *Oryza* species	24	Well-watered, drought	RCBD	3
2017	Screenhouse dry season (2017DS)	*Oryza glumaepatula* accessions	69	Well-watered, drought	RCBD	3
2017-2018	Screenhouse wet season (2017WS)	*Oryza glumaepatula* accessions	68	Well-watered, drought	RCBD	3

Supplemental Table S2. List of the accessions of *Oryza* species screened for root growth and drought response in greenhouse cylinder and screenhouse paddy experiments.

Number	Species	Greenhouse cylinder experiment	Screenhouse paddy experiment
1	O. alta acc. 105143	√	√
2	O. australiensis acc.100882	√	√
3	O. barthii acc. 100936	√	√
4	O. brachyantha acc. 101232	√	√
5	O. coarctata acc. 104502		√
6	O. echingeri acc. 101424		√
7	O. glaberrima acc. 96717 (CG14)	√	√
8	O. grandiglumis acc.101405	√	√
9	O. glumaepatula acc.105692	√	√
10	O. granulata acc.102118		
11	O. latifolia acc. 100914	√	√
12	O. longiglumis acc. 105148		√
13	O. longistaminata acc. 110404	√	√
14	O. meridionalis acc.105301	√	√
15	O. meyeriana acc.89241	√	
16	O. minuta acc. 101141		√
17	O. nivara acc. 80455	√	√
18	O. officinalis acc. 100896	√	√
19	O. punctata acc.105690		√
20	O. rhizomatis acc.105432	√	√
21	O. ridleyi acc 100821	√	√
22	O. rufipogon acc. 106424		√
23	O. sativa (IR64)	√	√
24	O. schlecteri acc. 82047		

Supplemental Table S3. Details of *O. glumaepatula* accessions evaluated in this study. NIG: National Institute of Genetics, IRRI: International Rice Research Institute.

No	Accession	Country	Seed Source
1	W1169	Cuba	NIG, Japan
2	W1171	Cuba	NIG, Japan
3	W1183	British Guiana	NIG, Japan
4	W1185	Suriname	NIG, Japan
5	W1187	Brazil	NIG, Japan
6	W1189	Brazil	NIG, Japan
7	W1191	Brazil	NIG, Japan
8	W1196	Colombia	NIG, Japan
9	W1477	Brazil	NIG, Japan
10	W2140	Brazil	NIG, Japan
11	W2145	Brazil	NIG, Japan
12	W2149	Brazil	NIG, Japan
13	W2160	Brazil	NIG, Japan
14	W2165	Brazil	NIG, Japan
15	W2173	Brazil	NIG, Japan
16	W2184	Brazil	NIG, Japan
17	W2192	Brazil	NIG, Japan
18	W2199	Brazil	NIG, Japan
19	W2203	Brazil	NIG, Japan
20	82031	Brazil	IRRI, Philippines
21	82032	Brazil	IRRI, Philippines
22	82033	Brazil	IRRI, Philippines
23	82034	Brazil	IRRI, Philippines
24	82035	Brazil	IRRI, Philippines
25	88793	Brazil	IRRI, Philippines
26	88794	Brazil	IRRI, Philippines
27	88796	Brazil	IRRI, Philippines
28	88797	Brazil	IRRI, Philippines
29	88798	Brazil	IRRI, Philippines
30	88799	Brazil	IRRI, Philippines
31	88800	Brazil	IRRI, Philippines
32	88801	Brazil	IRRI, Philippines
33	88802	Brazil	IRRI, Philippines
34	88803	Brazil	IRRI, Philippines
35	88804	Brazil	IRRI, Philippines
36	88805	Brazil	IRRI, Philippines
37	88806	Brazil	IRRI, Philippines
38	88807	Brazil	IRRI, Philippines
39	88808	Brazil	IRRI, Philippines
40	88810	Brazil	IRRI, Philippines
41	100184	Cuba	IRRI, Philippines
42	100894	NA	IRRI, Philippines
43	100924	Brazil	IRRI, Philippines
44	100968	Suriname	IRRI, Philippines
45	100969	Suriname	IRRI, Philippines
46	100970	Brazil	IRRI, Philippines
47	100971	Brazil	IRRI, Philippines
48	101960	Brazil	IRRI, Philippines
49	103812	Venezuela	IRRI, Philippines
50	104387	Brazil	IRRI, Philippines
51	105465	French Guiana	IRRI, Philippines
52	105561	Colombia	IRRI, Philippines
53	105661	Brazil	IRRI, Philippines
54	105662	Brazil	IRRI, Philippines
55	105663	Brazil	IRRI, Philippines
56	105665	Brazil	IRRI, Philippines
57	105666	Brazil	IRRI, Philippines
58	105667	Brazil	IRRI, Philippines
59	105668	Brazil	IRRI, Philippines
60	105670	Brazil	IRRI, Philippines
61	105672	Brazil	IRRI, Philippines
62	105686	Brazil	IRRI, Philippines
63	105687	Brazil	IRRI, Philippines
64	105688	Brazil	IRRI, Philippines
65	105689	Brazil	IRRI, Philippines
66	105692	Brazil	IRRI, Philippines
67	106232	Brazil	IRRI, Philippines
68	106233	Brazil	IRRI, Philippines
69	106243	Brazil	IRRI, Philippines

Supplemental Table S4. *Oryza glumaepatula* panel: Analysis of variance results of multiple phenotypic traits measured in dry and wet seasons for the *O. glumaepatula* accessions. The p values of all the factors are shown.

		**Dry season**				**Wet season**			
**Traits**	**Trait description**	**Block**	**Trt**	**Geno**	**Geno x Trt**	**Block**	**Trt**	**Geno**	**Geno x Trt**
**Shoot**									
SDW	Shoot dry weight	<0.001	<0.001	<0.001	0.05	0.02	<0.001	<0.001	0.25
PHT	Plant Height	0.02	<0.001	<0.001	<0.001	<0.001	<0.001	<0.001	0.001
TLN	Tiller Number	<0.001	<0.001	<0.001	<0.001	0.08	<0.001	<0.001	0.07
**Leaf**									
LWP	Leaf water potential	<0.001	<0.001	0.59	0.92	0.01	0.27	0.19	0.29
LOP	Leaf osmotic potential	0.002	<0.001	<0.001	0.46	<0.001	<0.001	<0.001	0.06
CHL	Chlorophyll content	<0.001	<0.001	<0.001	<0.001	0.05	<0.001	<0.001	<0.001
SLA	Specific leaf area	0.01	0.81	<0.001	0.49	0.002	<0.001	<0.001	0.02
**Root**									
RLD 0-15cm	Root length density 0-15 cm	0.003	<0.001	0.19	0.74	0.86	<0.001	0.09	0.52
RLD 15-30cm	Root length density 15-30 cm	0.21	<0.001	0.01	0.43	<0.001	<0.001	0.66	0.99
RLD 30-45cm	Root length density 30-45 cm	<0.001	<0.001	0.12	0.93	0.03	<0.001	0.71	0.35
RLD 45-60cm	Root length density 45-60 cm	<0.001	0.86	0.04	0.41	0.04	0.40	0.45	0.64
LRP 0-15cm	Lateral root percentage 0-15 cm	0.05	<0.001	0.37	0.42	<0.001	<0.001	0.52	0.39
LRP 15-30cm	Lateral root percentage 15-30 cm	0.53	<0.001	0.10	0.03	0.66	<0.001	0.65	0.29
LRP 30-45cm	Lateral root percentage 30-45 cm	0.03	<0.001	0.01	0.14	<0.001	<0.001	0.99	0.79
LRP 45-60cm	Lateral root percentage 45-60 cm	0.01	<0.001	0.29	0.04	0.80	<0.001	0.66	0.68
RDW 0-15cm	Root dry weight 0-15 cm	0.45	<0.001	<0.001	0.31	0.60	<0.001	<0.001	0.02
RDW 15-30cm	Root dry weight 15-30 cm	0.65	<0.001	0.27	0.55	0.44	0.01	0.50	0.98
RDW 30-45cm	Root dry weight 30-45 cm	0.32	0.001	0.22	0.54	0.01	0.03	0.21	0.78
RDW 45-60cm	Root dry weight 45-60 cm	0.09	0.01	<0.001	0.16	0.16	0.18	0.57	0.71
TRD	Total root dry weight	0.05	<0.001	0.001	0.95	0.32	<0.001	0.002	0.07
CRN	Crown root number	0.95	<0.001	<0.001	0.28	0.57	<0.001	0.003	0.85

Supplemental Table S5. Summary of data for each *O. glumaepatula* accession in this study by Illumina resequencing to a coverage of 3x: sequencing depth, and proportion of reads mapped.

SampleID	AccessionNo	MeanCoverage
1	104387	3.98499192
2	105561	3.946993317
3	88800	4.589413249
4	103812	4.760786668
5	100894	4.484162402
6	106243	4.44804086
7	105661	5.169863089
8	W2203	4.444536837
9	82034	4.140329776
10	105662	5.001557059
11	W1187	4.638687539
12	88796	4.890336008
13	82035	4.223367358
14	88799	5.735912975
15	W1189	3.285314705
17	W2140	4.031644845
18	82031	4.50709561
19	106233	3.71771759
20	100184	4.099008652
21	105465	3.978736185
22	88798	4.055805678
23	W1185	4.138896369
24	105686	4.182482973
25	100970	4.164384784
26	W2192	3.289206716
27	101960	3.291290151
28	105666	3.051120989
29	W2199	3.945130619
30	88810	4.583081543
31	105689	4.554388449
32	88804	4.841096564
33	W2173	4.607271652
34	82033	3.791052155
35	88807	3.322846042
36	88802	3.864511003
37	106232	3.220952597
38	W2160	3.459366182

SampleID	AccessionNo	MeanCoverage
39	100971	3.436252847
40	W2184	4.021300494
41	88801	3.65587407
42	105672	0.893737478
44	88794	14.57507069
45	W2145	3.724174963
46	105668	3.057737737
47	88803	4.144111951
48	88797	2.990158227
49	105687	3.079093195
50	W2165	3.665904153
51	105670	3.163676281
52	100969	3.545280432
53	W1477	3.282387582
54	82032	3.146027125
56	W1196	3.071959698
57	88805	3.137157764
58	105688	3.676617822
59	88808	4.089399179
60	W1171	3.788020002
61	W1191	3.603497153
62	100924	3.475823493
63	100968	3.596532627
64	105665	6.929211554
65	105663	3.440914842
66	W1169	6.419809712
67	105692	4.595342015
68	105667	3.86984594
69	88806	3.898974287
70	W2149	4.040058694
72	W1183	4.158521447

Supplemental Table S6. Statistics of different assemblies before and after GPM editing.

	CANU	FALCON		MECAT	OgluRS3	GenBank AC# ALNU03000000			
File name	canu1st.contigs.fasta	p_ctg	a_ctg	O_glumaepatula.contigs.fasta	O_glumaepatula_canu-GPM-ctg-polish2nd-000887.fa	Chr	ChrUn	Chloroplast	Mitochondrion
Number of contigs	94	4266	202	350	87	33	35	8	11
Total size of contigs	3.9E+08	4.9E+08	1.1E+07	3.9E+08	3.9E+08	3.9E+08	2412673	815407	1377910
Longest contig	3.7E+07	2858744	159419	1.2E+07	3E+07	3E+07	205312	194041	257905
Shortest contig	18091	38	5247	35031	18091	408392	18091	64741	75650
Number of contigs > 500 nt	94 (100.0%)	4262 (99.9%)	202 (100.0%)	350 (100.0%)	87 (100.0%)	33	35	8	11
Number of contigs > 1K nt	94 (100.0%)	4256 (99.8%)	202 (100.0%)	350 (100.0%)	87 (100.0%)	33	35	8	11
Number of contigs > 10K nt	94 (100.0%)	4001 (93.8%)	199 (98.5%)	350 (100.0%)	87 (100.0%)	33	35	8	11
Number of contigs > 100K nt	53 (56.4%)	831 (19.5%)	19 (9.4%)	276 (78.9%)	46 (52.9%)	33	5	3	5

Number of contigs > 1M nt	37 (39.4%)	79 (1.9%)	0 (0.0%)	107 (30.6%)	32 (36.8%)	32	0	0	0
Mean contig size	4126787	114145	55493	1103930	4491778	1.2E+07	68933	101925	125264
Median contig size	118410	37258	46205	417381	103477	9376969	60091	96420.5	97021
N50 contig length	1.5E+07	441319	62345	2830672	1.6E+07	1.8E+07	70339	96803	108127
L50 contig count	9	288	62	37	9	8	12	4	4
N50 contig - NG50 contig length difference	1.5E+07	441319	62345	2830672	1.6E+07	-	-	-	-
contig %A	27.96	27.85	27.26	28	27.96	-	-	-	-
contig %C	22.03	22.13	22.69	22.02	22.04	-	-	-	-
contig %G	22.04	22.14	22.6	22.01	22.03	-	-	-	-
contig %T	27.97	27.88	27.44	27.98	27.97	-	-	-	-

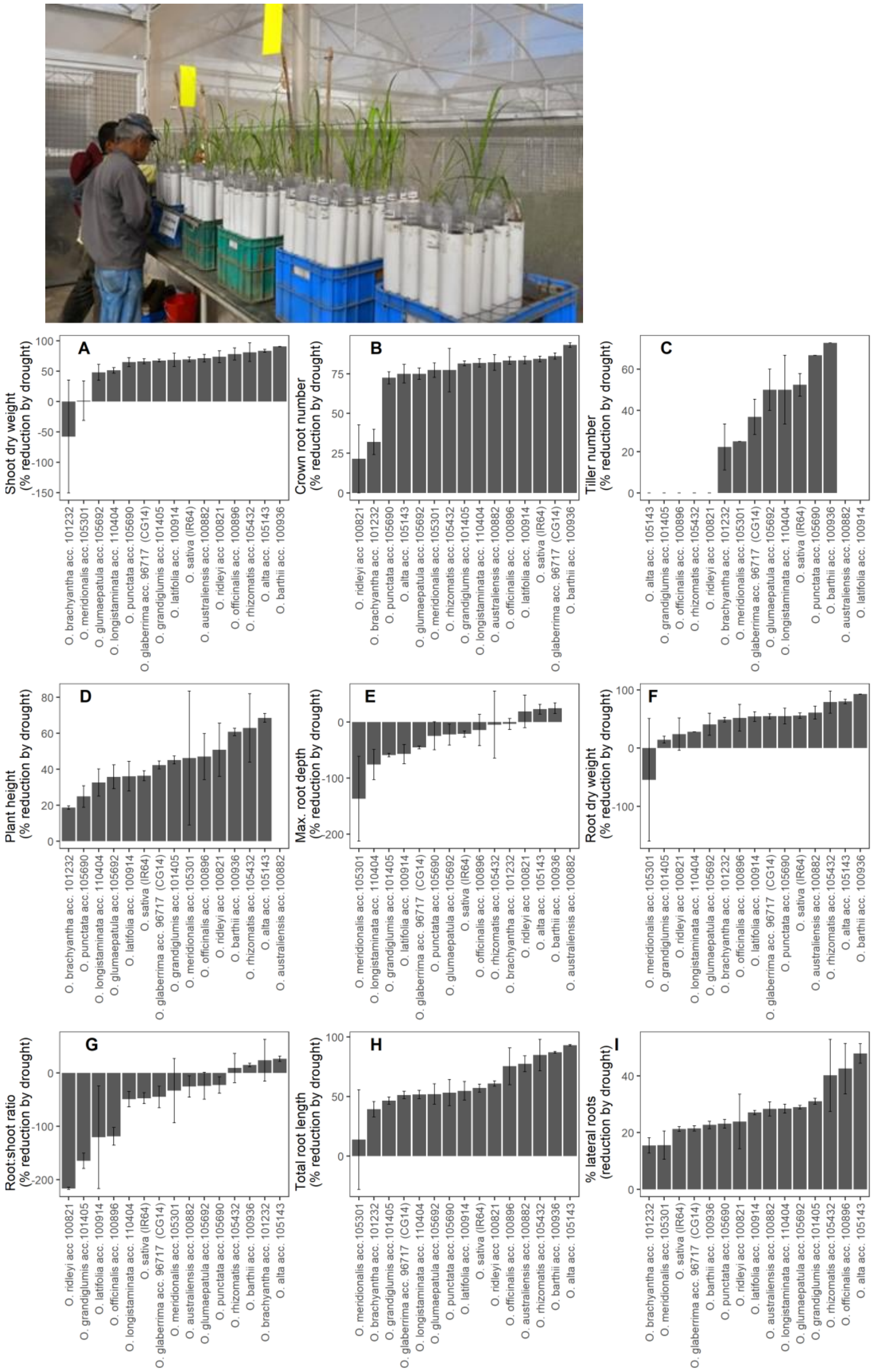

Supplemental Fig. S1. Screening of 24 *Oryza* species in a greenhouse cylinder study at IRRI during the 2015 dry season. The reduction of A) shoot biomass, B) tiller number, C) plant height, D) root:shoot ratio, E) total root length, F) root dry weight, G) crown root number, H) max. root depth, and I) % lateral roots in the drought stress treatment as compared to the well-watered treatment. Values shown are means ± standard error (n=4).

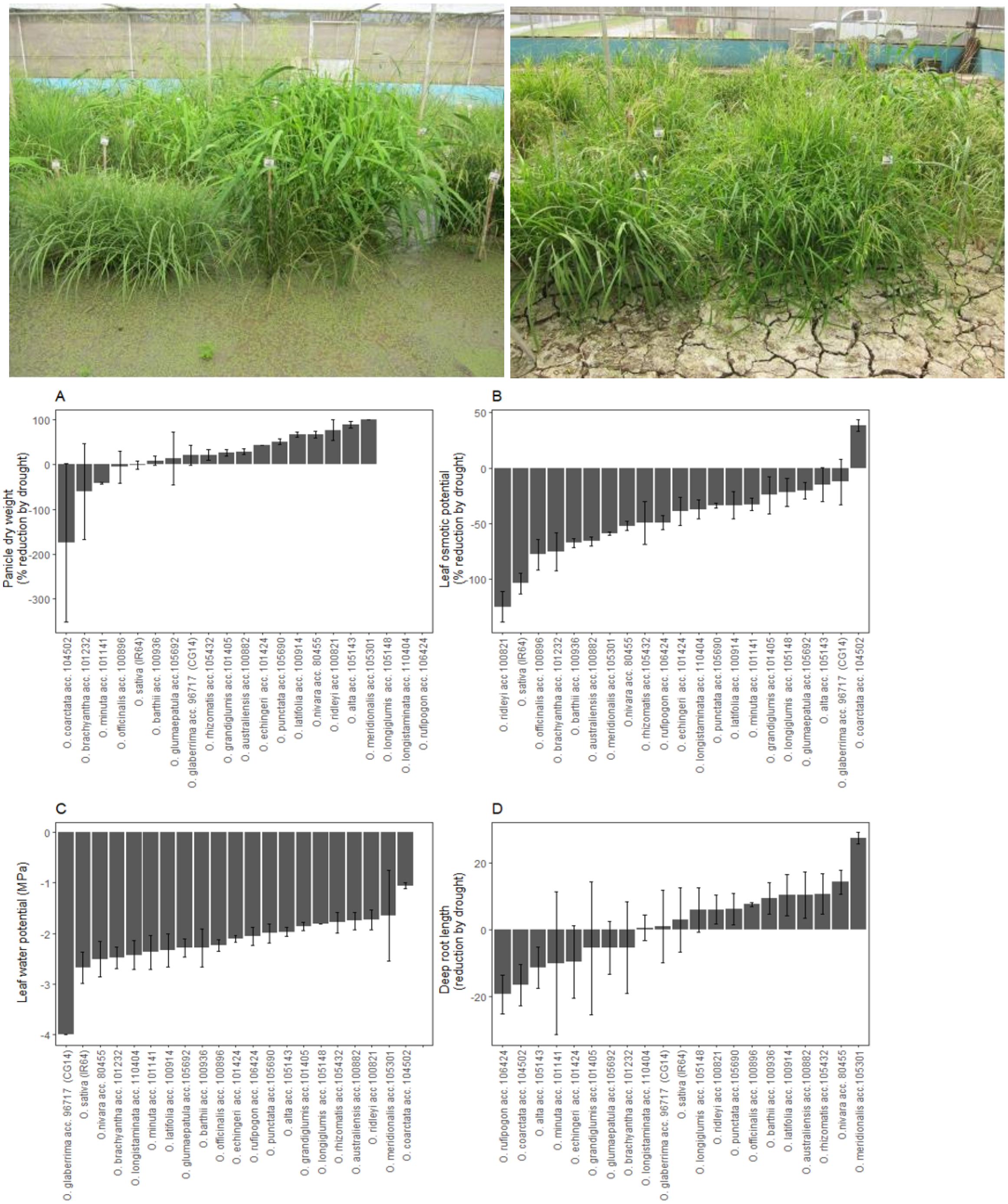

Supplemental Fig. S2. Screening of 24 *Oryza* species in a screenhouse paddy study at IRRI during the 2016 dry season. Top: The left side is the well-watered condition and the right side is the drought stress condition. Bottom: Response of 24 *Oryza* species to drought in the screenhouse paddy experiment. A) the reduction of panicle dry weight, B) the reduction of leaf osmotic potential, C) leaf water potential and D) the reduction of deep (>30 cm) root length in the drought stress treatment as compared to the well-watered treatment. Values shown are means ± standard error (n=3).

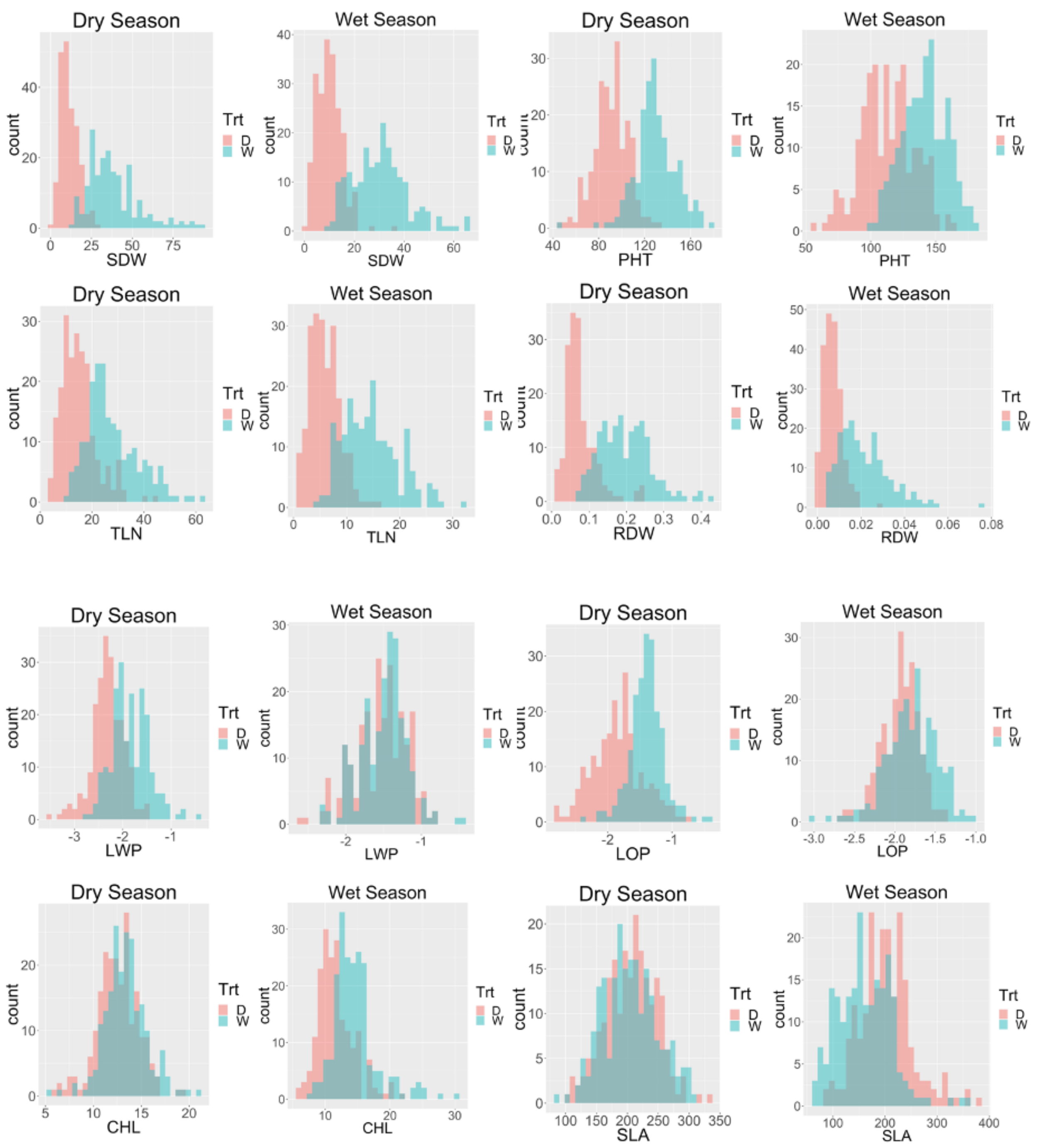

Supplemental Fig. S3 Histograms of shoot traits measured on the *O. glumaepatula* panel under well-watered (W) and drought stressed (D) conditions in both seasons. See Supp. Table S4 for trait names and the treatment and genotype effects on each trait.

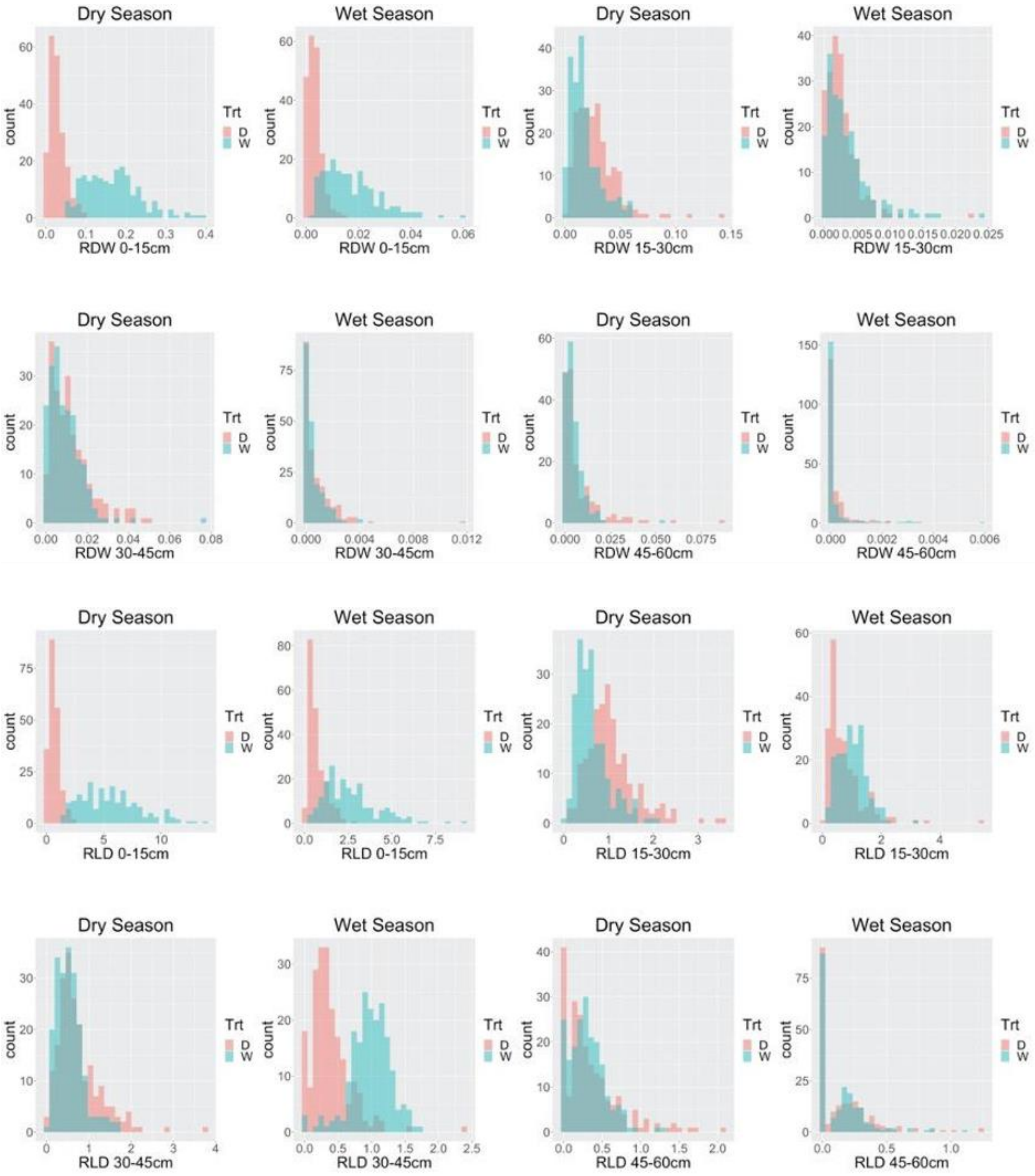

Supplemental Fig. S4 Histograms of root length density (cm cm^{-3}) and root dry weight within each depth increment measured on the *O. glumaepatula* panel under well-watered (W) and drought stressed (D) conditions in both seasons. See Supp. Table S4 for trait names and the treatment and genotype effects on each trait.

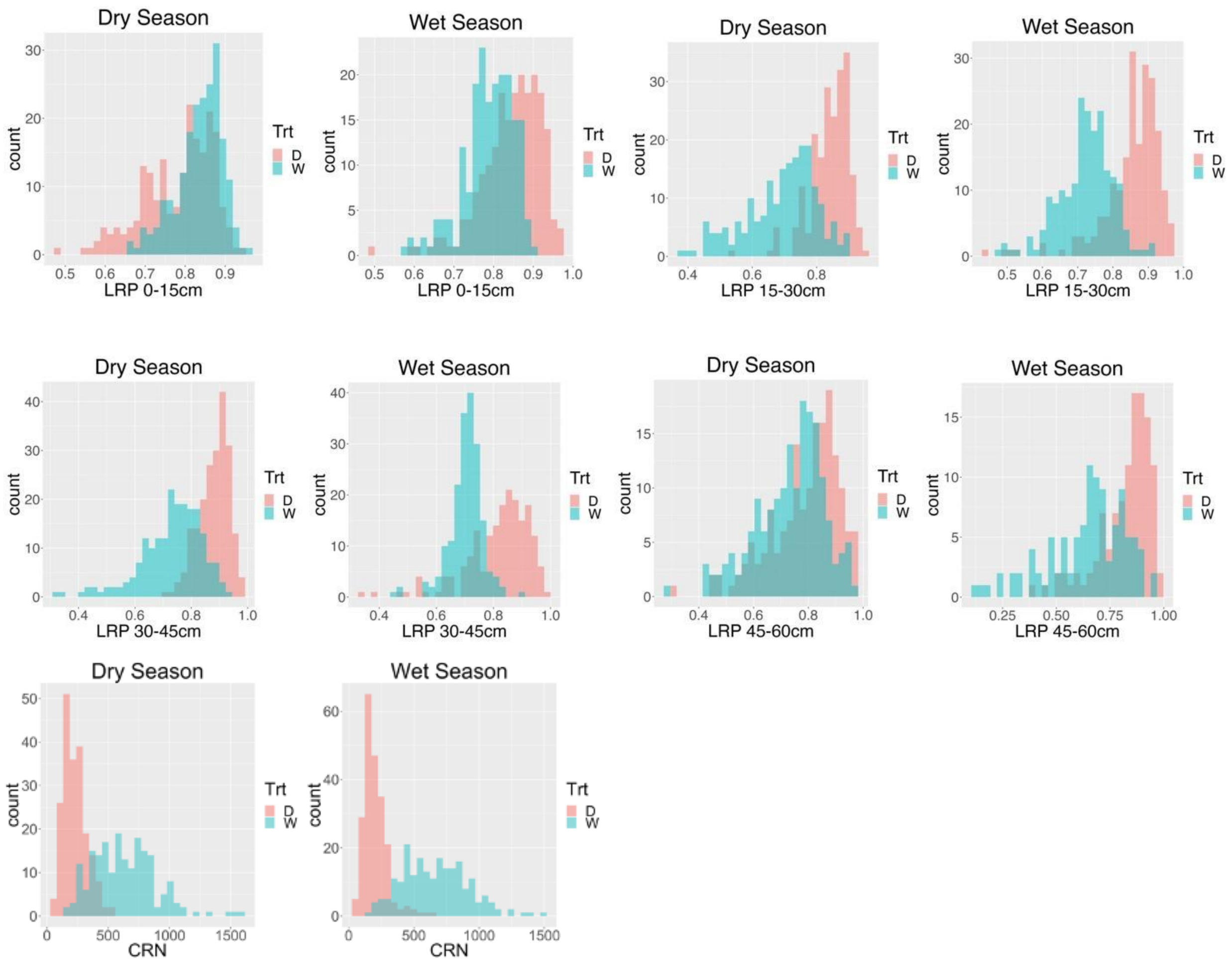

Supplemental Fig. S5 Histograms of the percent of total root length as lateral roots within each depth increment and crown root number measured on the *O. glumaepatula* panel under well-watered (W) and drought stressed (D) conditions in both seasons. See Supp. Table S4 for trait names and the treatment and genotype effects on each trait.

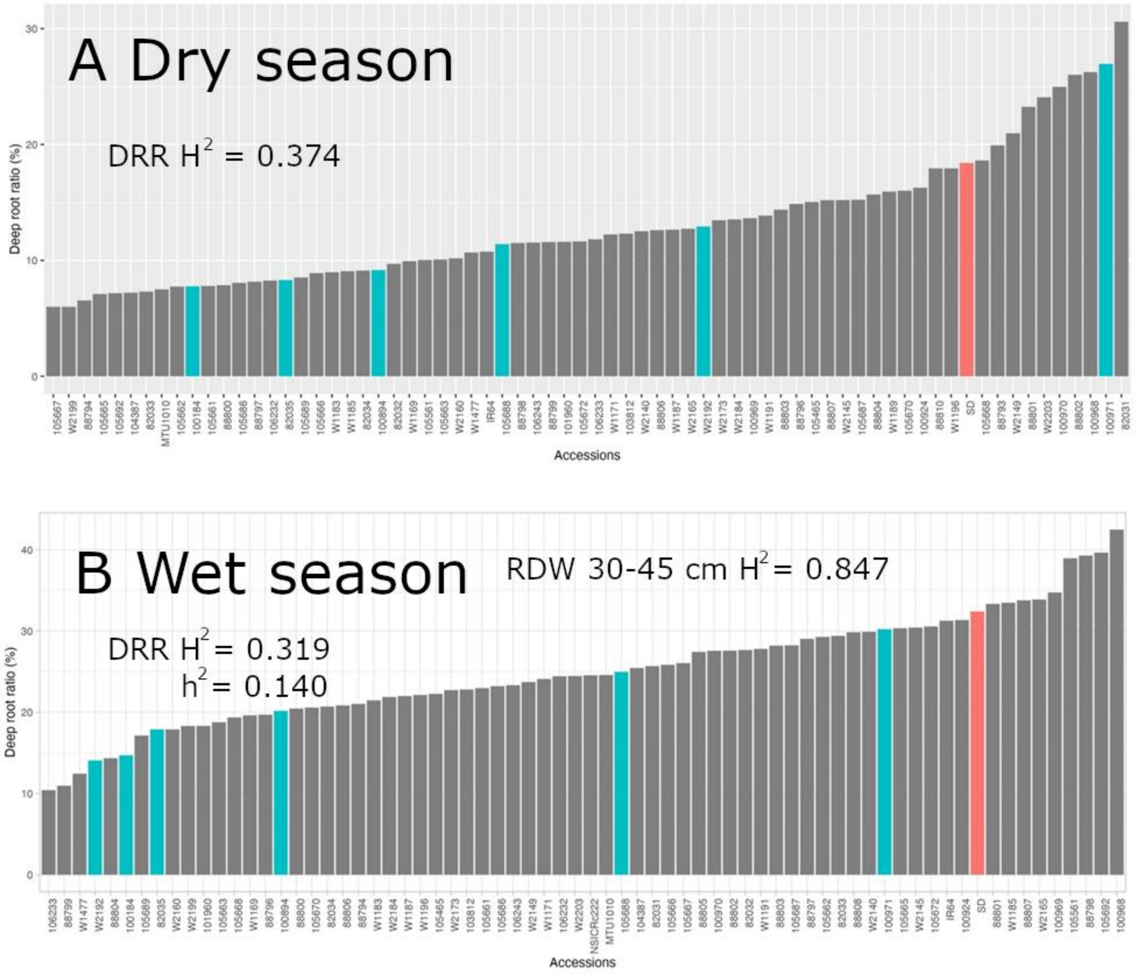

Supplemental Fig. S6. Rank order plot of the proportion of deep roots under well-watered conditions. All 69 *O. glumaepatula* accessions along with the *O. sativa* checks are shown in the dry (A) and wet (B) season experiments. The potential donors are highlighted in blue and the drought tolerant check Sahbhagi dhan in red. Any non-zero broad sense (H^2, based on phenotypic data) and narrow sense (h^2, based on genomic data) heritability values are indicated for the relevant root traits (RDW: root dry weight, DRR: deep root ratio [$RLD_{30\text{-}60\ cm}/RLD_{0\text{-}60\ cm}$ x 100]) in the well-watered treatment. Values shown are means (n=3).

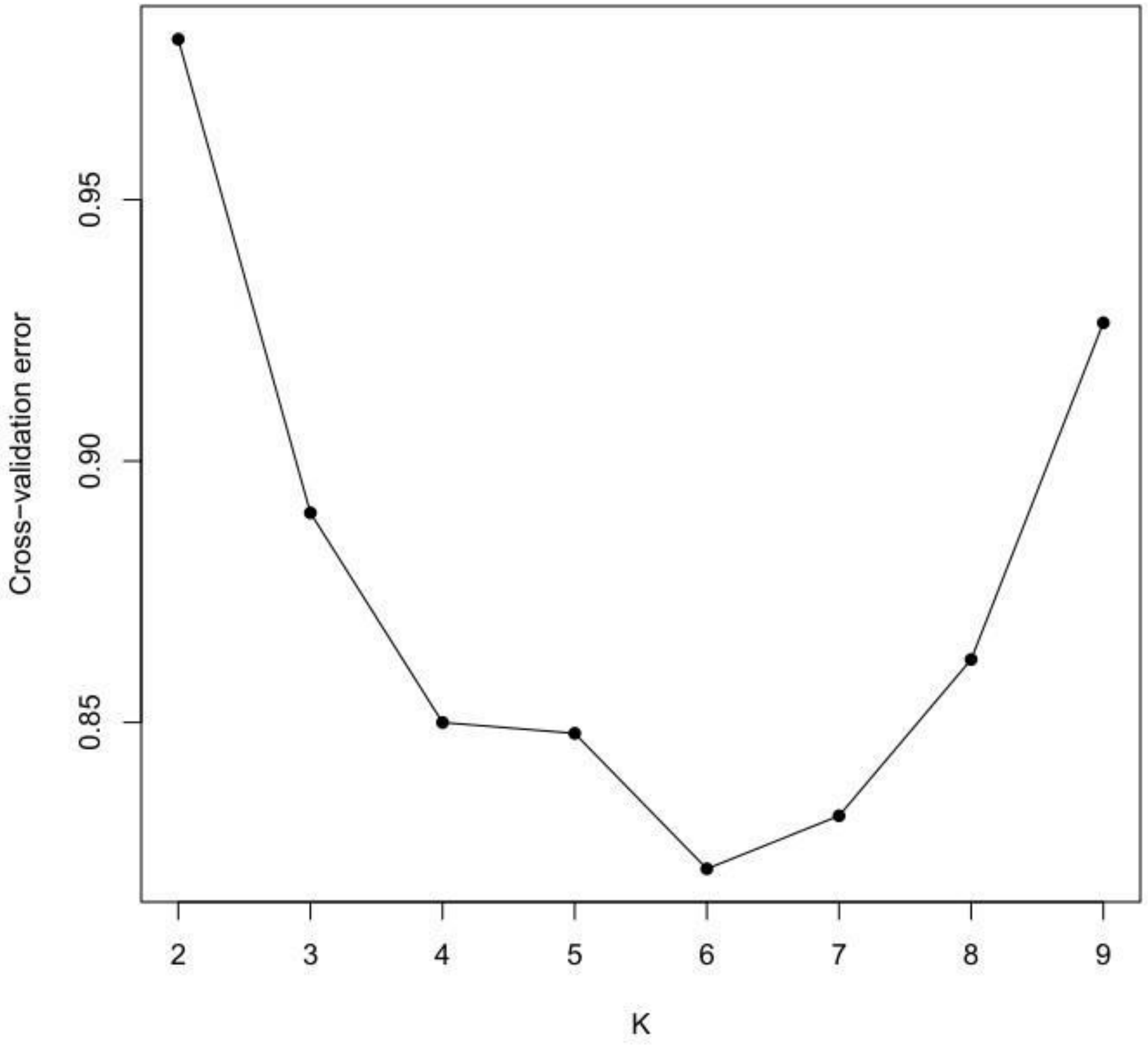

Supplemental Fig. S7. CV error plot for selection of the "best K" for the population structure analysis shown in Fig. 6.

A

Sample ID	*O. glumaepatula* (OgluRS3.ALNU03)	*O. sativa* (Nipponbare IRGSP1.0)
Sample_OG_1	95.09%	96.82%
Sample_OG_2	95.31%	94.04%
Sample_OG_20	90.53%	92.17%
Sample_OG_26	95.76%	97.62%
Sample_OG_35	96.48%	97.91%

B

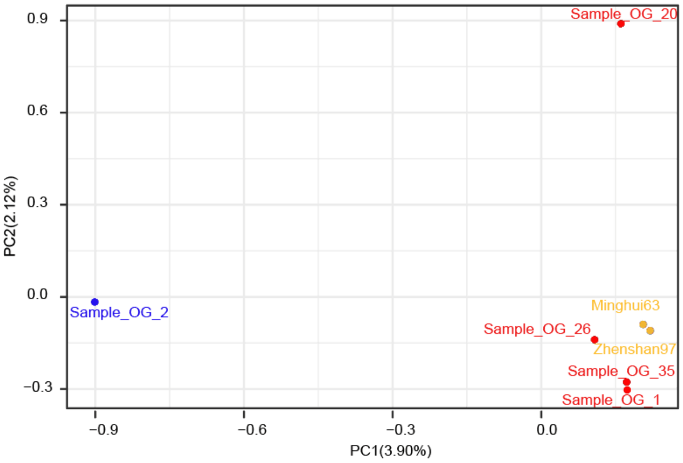

C

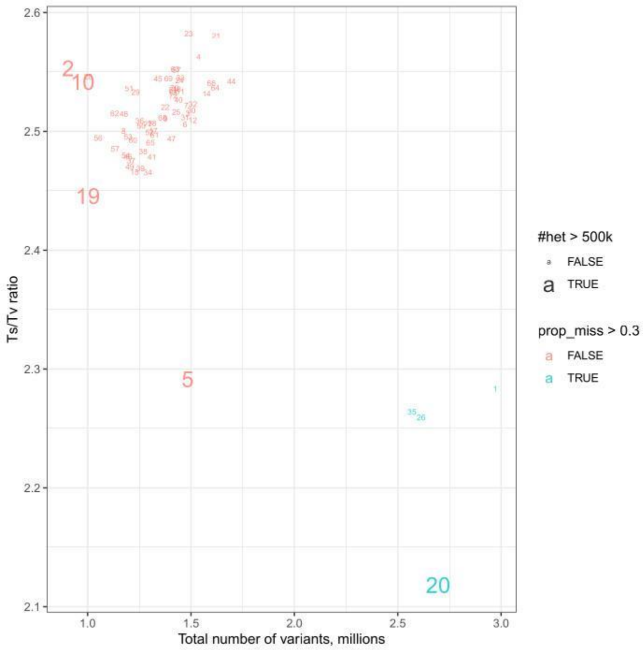

D

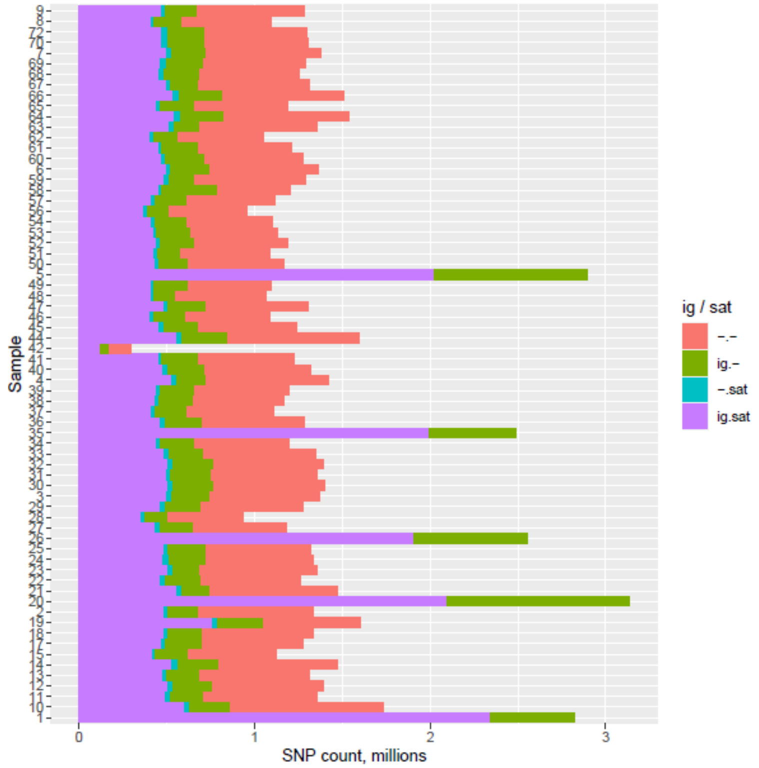

E

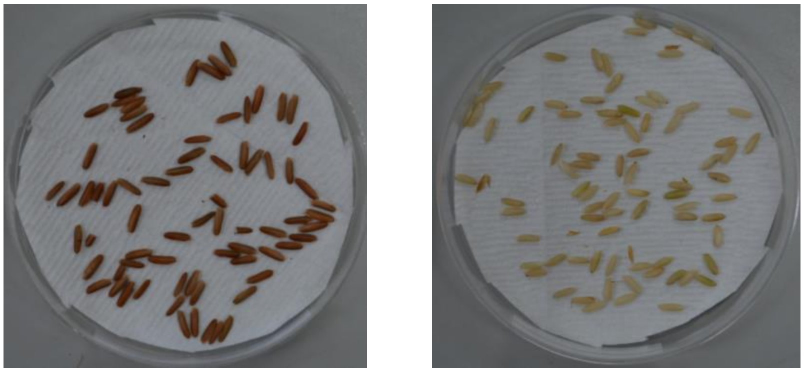

Supplemental Fig. S8. Dissecting the genetic identity of the four Cluster 1 accessions. A) Mapping of resequencing reads of the four Cluster 1 accessions (as well as *O. glumaepatula* sample_OG_2 as a positive control) to both *O. glumaepatula* (OgluRS3.ALNU03) and *O. sativa* (Nipponbare IRGSP1.0) reference genomes. Accessions are identified by VCF ID as defined in Fig. 6. B) PCA clustering analysis carried out after aligning the resequencing reads of the five *O. glumaepatula* accessions to the *O. glumaepatula* genome by using GATK for SNP calling. Compared with the *O. glumaepatula* accession OG_2, the number of SNPs in the four accessions of interest is significantly higher, indicating that they are farther from *O. glumaepatula*. The Cluster 1 accessions are grouped with the two *O. sativa* accessions (Zhenshan 97 & Minghui 63), but far from the *O. glumaepatula* sample_OG_2. C) Ts/Tv ratio. TS: the number of transitions. Tv: the number of transversions. The four accessions with a high proportion of Cluster 1 showed a high number of variants and markedly different Ts/Tv ratios from the rest of the panel, as well as an elevated proportion of missing calls which is consistent with having large introgressions from species other than *O. glumaepatula*. The numbers are VCF ids of the samples. Color highlights the samples with many missing calls. D) SNP sharing analysis using several *O. sativa* samples from the 3K set (two indica samples LIMA and MH63 and one aus NATEL BORO) used in Zhou et al. 2020 showed indeed a large SNP sharing with *O. sativa* across all genetic outlier samples. Colored bars indicate SNPs appearing in introgressed/Cluster 1 lines (ig) and SNPs appearing in *O. sativa* lines (sat), SNPs appearing in both *O. sativa* and introgressed/Cluster 1 lines (ig/sat), and neither *O. sativa* nor in introgressed lines (-/-). E) Example images of the seed types of the potential donors (left: accession 100941, right: accession 100894).

Zhou Y, Chebotarov D, Kudrna D, Llaca V, Lee S, Rajasekar S, Mohammed N, Al-Bader N, Sobel-Sorensen C, Parakkal P, et al. (2020) A platinum standard pan-genome resource that represents the population structure of Asian rice. Sci Data **7**, 113 https://doi.org/10.1038/s41597-020-0438-2

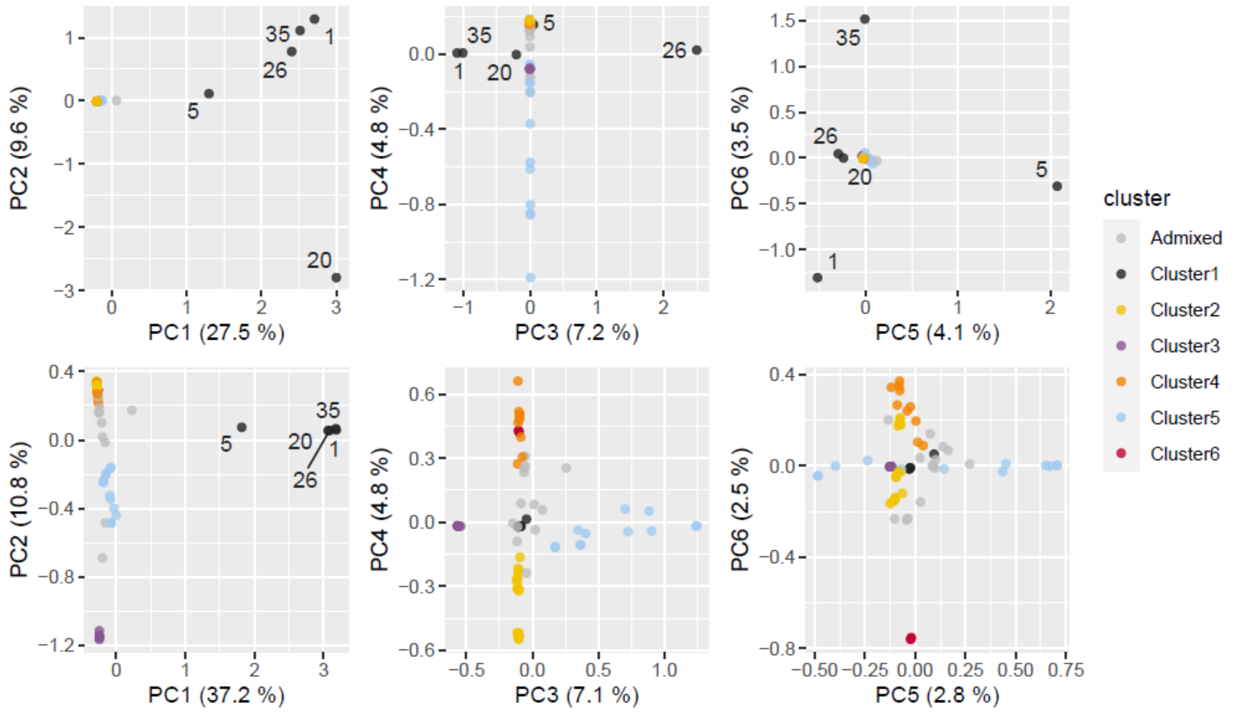

Supplemental Fig. S9. Comparison of PCAs with and without different levels of filtering. Panels in the top row were generated with no MAF filtering, and panels in the bottom row were generated with MAF filtering as in our manuscript (~0.05). The columns from left to right show PC1-2, PC3-4, and PC5-6. Points are colored by the clusters determined by ADMIXTURE analysis.

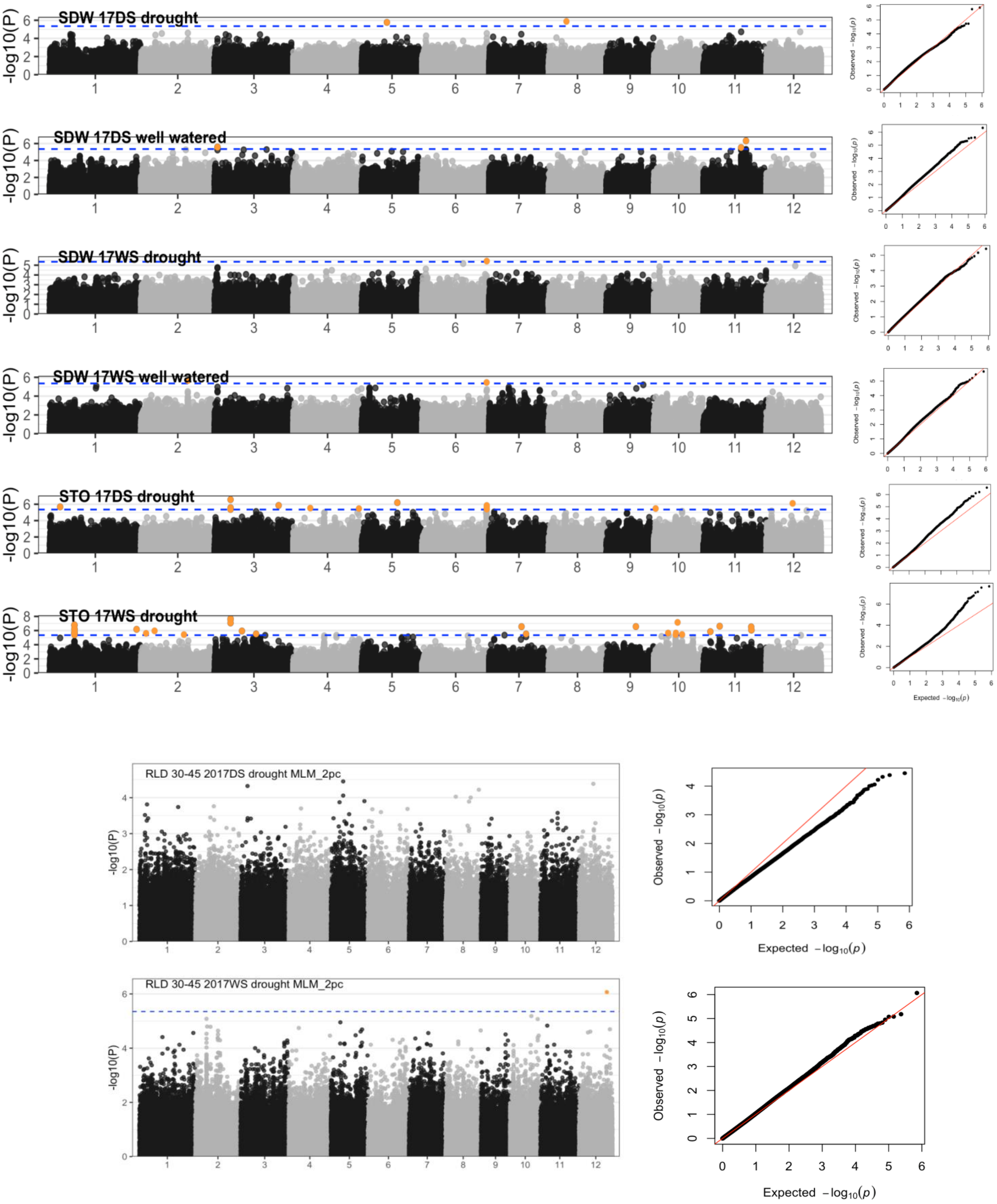

Supplementary Fig. S10. Manhattan plots and QQ plots for shoot dry weight, stomatal density, and root length density at the 30-45 cm depth. Co-locating GWAS peaks are summarized in Table 2.

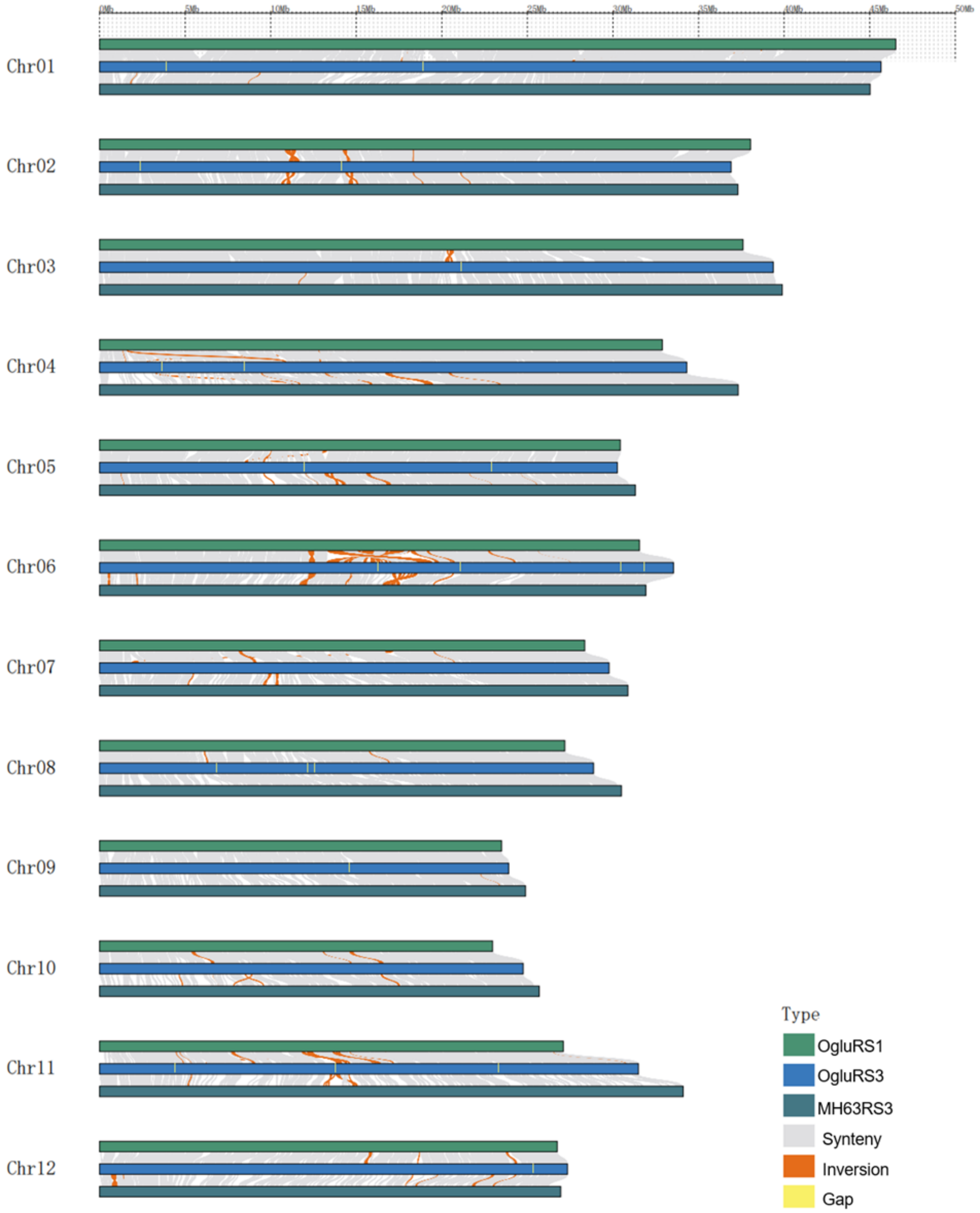

Supplemental Fig. S11. Collinearity of the *O. glumaeptaula* reference genome assembly with the Minghui 63 gap-free assembly MH63RS3.

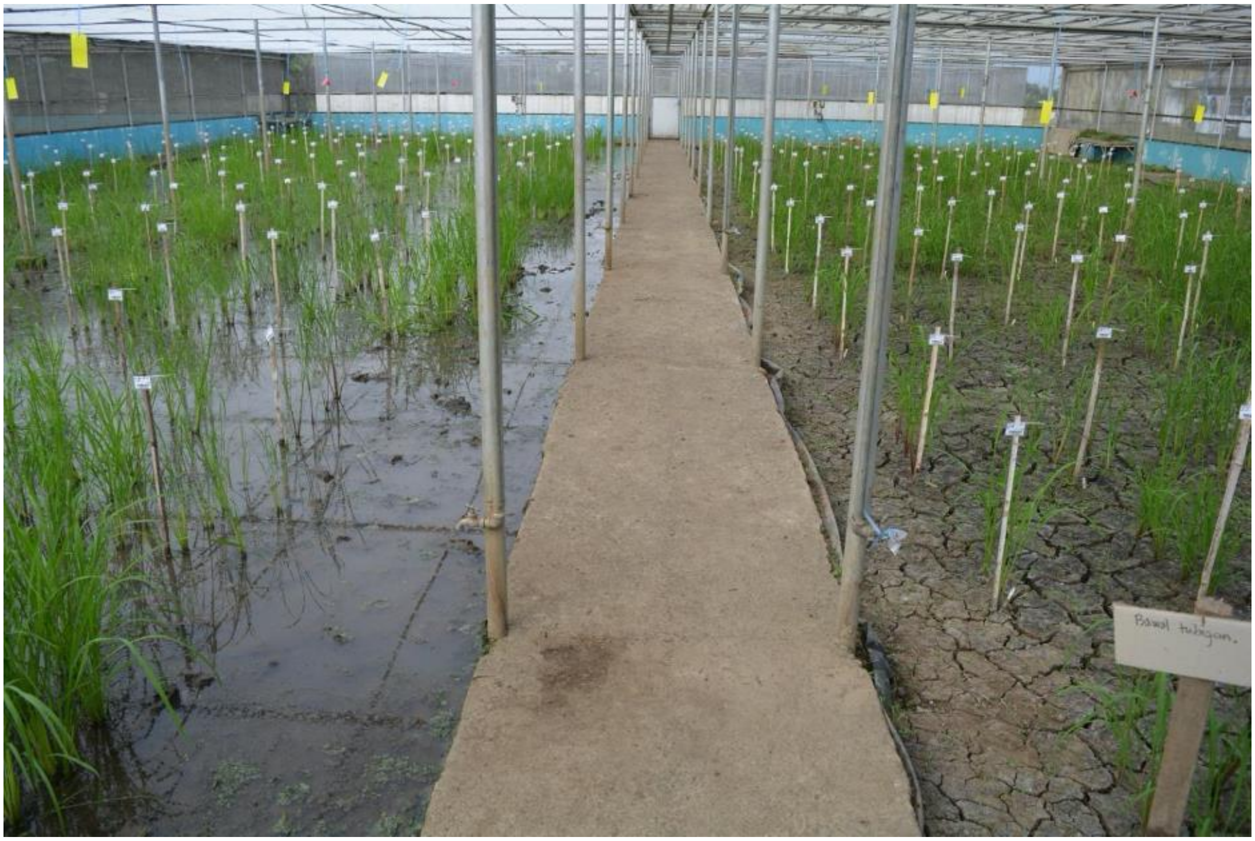

Supplemental Fig. S12. Screening of *O. glumaepatula* accessions in the screenhouse facility at IRRI during the 2017 dry season experiment. The left side is the well-watered condition and the right side is the drought stress condition.

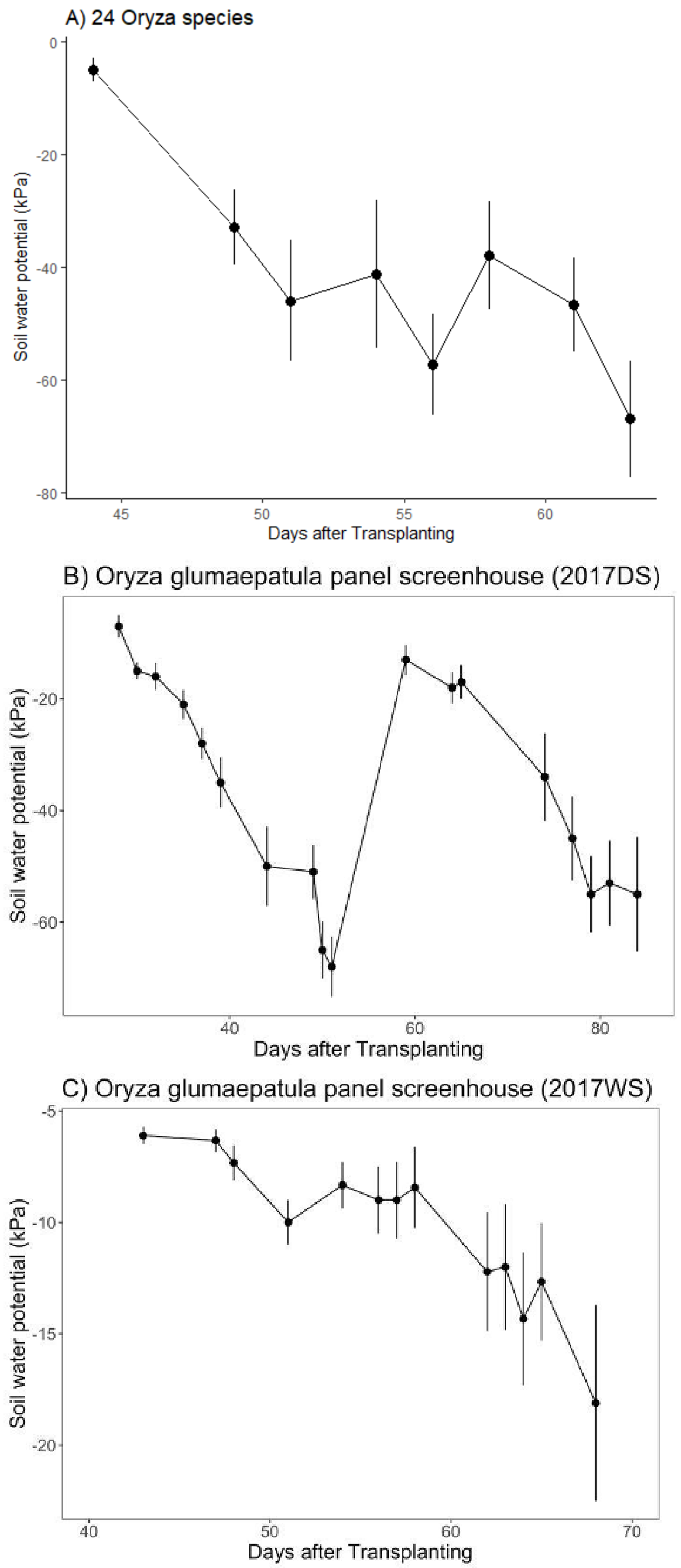

Supplemental Figure S13. Soil water potential readings from a soil depth for 30 cm using tensiometers in the screenhouse experiments. Values shown are means (n=3) in A) the 24 *Oryza* species and B-C) the 2017DS-2017WS *O. glumaepatula* panel experiments.
